# Physics-informed differentiable solvers for learning parametric solution manifolds in heterogeneous physical systems

**DOI:** 10.1093/pnasnexus/pgag195

**Published:** 2026-06-01

**Authors:** Milad Panahi, Giovanni Michele Porta, Monica Riva, Alberto Guadagnini

**Affiliations:** Dipartimento di Ingegneria Civile e Ambientale, Politecnico di Milano, Piazza L. da Vinci 32, Milano 20133, Italy; Dipartimento di Ingegneria Civile e Ambientale, Politecnico di Milano, Piazza L. da Vinci 32, Milano 20133, Italy; Dipartimento di Ingegneria Civile e Ambientale, Politecnico di Milano, Piazza L. da Vinci 32, Milano 20133, Italy; Dipartimento di Ingegneria Civile e Ambientale, Politecnico di Milano, Piazza L. da Vinci 32, Milano 20133, Italy

**Keywords:** physics-informed neural networks, parameterized PDEs, differentiable physics, uncertainty quantification, Darcy flow

## Abstract

Quantifying parametric uncertainty in partial differential equations is a central challenge to our ability to model the behavior of heterogeneous systems. This challenge is relevant to a variety of fundamental and application-oriented implications where system properties exhibit significant (and often uncertain) spatial heterogeneity. We address this by reformulating a physics-informed neural network as a differentiable solver that learns the continuous solution manifold for steady-state Darcy flow. Our framework requires only a single training run, circumventing the need for costly retraining for each new parameter instance. The approach is demonstrated through two representations of spatially heterogeneous hydraulic conductivity fields: a direct analytical form and a novel data-driven formulation resting on an autoencoder to create a low-dimensional latent encoding. A key innovation is the integration of the differentiable decoder into the physics-informed loss function, enabling on-the-fly reconstruction of complex conductivity fields. The approach yields accurate, mass-conserving flow solutions and supports efficient uncertainty quantification, providing a general methodology for physics-constrained data-driven modeling of heterogeneous systems.

Significance statement Modeling the behavior of complex physical systems, from groundwater flow to material science, is often hindered by the high computational cost of solving the governing parameterized equations for every new set of conditions. We overcome this bottleneck by framing a physics-informed neural network as a differentiable solver capable of learning the entire family of solutions in a single training run. Our framework uniquely integrates a differentiable generative model directly into a physics-informed loss, enabling it to solve systems with complex, spatially heterogeneous properties. We demonstrate that this purely physics-trained model robustly preserves physical principles such as local mass conservation. This work provides a flexible and efficient pathway for building physically consistent digital twins for uncertainty quantification in a broad range of applications.

## Introduction

Many scientific and engineering systems are governed by partial differential equations (PDEs) involving uncertain and spatially heterogeneous parameters, whose characterization is critical for prediction, control, and decision-making under uncertainty. Thus, a central challenge is to develop computational frameworks that can efficiently propagate high-dimensional uncertainty while retaining consistency with the underlying physical laws. In several domains, ranging from climate science to hydrogeology and materials science, governing equations embed system parameters that exhibit strong spatial variability (1, 2). A representative example is subsurface fluid flow, where hydraulic conductivity K(x) can span several orders of magnitude and display complex multiscale spatial organization, strongly influencing system behavior and the emergence of preferential flow pathways (3–6). Accurately quantifying uncertainty in such parameters and propagating it through predictive models is central to reliable risk assessment and uncertainty-aware decision-making (7–9).

Traditional approaches (typically framed, eg in a Monte Carlo-based strategy (10)) rely on ensembles of forward simulations that are usually obtained through numerical solution of a system model. While robust, these methods become computationally prohibitive as dimensionality of the parameter space increases. To alleviate this burden, surrogate modeling techniques such as, eg polynomial chaos expansions and Gaussian process regression (11) have been developed to approximate the response of full (high-fidelity) models. Perturbation-based approaches (5, 12–15) yield theoretically rigorous estimates of statistical moments and spatial correlations of state variables, such as hydraulic head and flow velocities. However, they are often restricted to simplified geometries or low-dimensional parameterizations and may be limited in adequately capturing strongly nonlinear dynamics or the effects of pronounced and complex spatial heterogeneity. These constraints motivate the development of flexible and scalable approaches for uncertainty quantification in complex systems.

Recent advances in machine learning have introduced new paradigms for modeling physical systems, including data-driven and hybrid approaches that combine physical knowledge with learned representations (16–20). Neural operator learning has emerged as a powerful framework for approximating mappings between parameter fields and PDE solutions (21), architectures such as Fourier neural operators and DeepONets enabling rapid predictions across a range of input configurations (22, 23). Despite their effectiveness, these approaches typically rely on supervised training using datasets generated from numerical simulations or observations. Physics-informed neural networks (PINNs, 24) offer an alternative upon embedding governing equations directly into the training process, enabling the solution of differential equations without labeled data. However, PINN formulations are typically designed to address individual PDE instances, or to infer unknown parameter values in the presence of labeled data. Hence, they do not explicitly tackle families of PDEs characterized by high-dimensional parameter spaces, particular those arising in the presence of spatially heterogeneous coefficients. Extensions such as physics-informed neural operators (PINOs, 25) combine operator learning with physics-based constraints. As such, they provide a trade-off between parametric generalization and solution-specific physical consistency. Despite these advances, no existing approach simultaneously provides a physics-driven solution framework that (i) operates without reliance on training data, (ii) generalizes across high-dimensional, spatially heterogeneous parameter fields, and (iii) yields a fully differentiable parameter-to-solution mapping suitable for sensitivity analysis and uncertainty quantification.

In this work, we introduce a differentiable, physics-driven framework that bridges this gap by unifying parametric PDE solving and uncertainty quantification within a single model. The proposed approach is formulated as a self-contained, PINN-based forward solver that approximates the solution of Darcy flow across both spatial and parametric domains, where uncertain parameters are represented as spatially heterogeneous fields. A central aspect of the framework is the construction of a continuous and fully differentiable parameter-to-solution mapping, enabling the direct evaluation of spatial derivatives via automatic differentiation (AD). This capability is essential for computing derived physical quantities, such as flow velocities, and for enabling gradient-based sensitivity analysis and uncertainty quantification. More broadly, this formulation contributes to the emerging paradigm of differentiable physics (26, 27), where physical models are embedded within end-to-end trainable systems.

We build on our previous work on PINN-based uncertainty quantification (PINN-UU) (28) and provide a significant extension of the framework to settings where uncertain parameters are represented as spatially varying fields rather than spatially uniform quantities. This generalization is achieved while preserving full differentiability of the parameter-to-solution mapping, thus enabling treatment of high-dimensional heterogeneous parameter spaces. We apply the proposed framework to steady-state Darcy flow, where the parameter vector encodes spatially heterogeneous hydraulic conductivity fields. We achieve our objective through integration of latent representations for random parameter fields, where the parameter vector encodes a heterogeneous field in a low-dimensional space, through generative models such as variational autoencoders (AEs) (29). AEs have been widely employed in the literature of reduced order modeling for uncertainty quantification (30, 31). The integration with our PINN-based approach requires differentiating through the decoding map that reconstructs the full spatial field, thereby embedding also this component within the broader paradigm of differentiable physics.

The key contributions of this study are summarized as follows:

- We develop a PINN-based parametric solver for flow in spatially heterogeneous media, leveraging an architecture inspired by PirateNets (32) to enhance training stability and efficiency. The method enables accurate prediction of both hydraulic head and derived velocity fields.
- We introduce a framework capable of handling uncertain input parameters characterized by prescribed distributions, supporting applications such as forward uncertainty quantification, sensitivity analysis, and experimental design. Two strategies are explored for constructing the parameter-to-solution mapping: (i) direct functional parameterization and (ii) latent representations learned through coordinate-based AEs.
- We address input spaces combining spatial coordinates and parameter domains through a multistage transfer learning strategy, improving convergence and robustness across heterogeneous conductivity fields.

The remainder of the article is organized as follows. The Methodology: differentiable physics-informed learning framework section presents the mathematical formulation and methodology. The Results and discussion section discusses the numerical results. The Conclusions and future perspectives section concludes the article and outlines future research directions.

## Methodology: differentiable physics-informed learning framework

Here, we illustrate our differentiable physics-informed learning framework. The neural network architecture and embedded physical constraints are integrated into a single computational graph, enabling simultaneous adherence to data and physics-constrained optimization via gradient-based methods. We detail the formal problem statement, the physics-informed learning framework, our strategies for encoding spatially heterogeneous conductivities, and the training approach.

### A general framework for parameterized PDEs

Let the spatial domain be an open set $\Omega \subset R^{d}$. The PDE system governing the system behavior is characterized by a vector of parameters λ of dimension $d_{\lambda }$ defined within a parameter space $\Lambda \subset R^{d_{\lambda }}$.

The relationship between the parameters and the solution is implicitly defined by a system of differential operators. We define an operator $F[u(x,\lambda )]=F_{x,\lambda }(u)$ that represents the governing equations within the spatial domain *Ω* and the parameter space *Λ*, and an operator $B[u(x,\lambda )]=B_{x,\lambda }(u)$ for the boundary conditions on ∂Ω. The vector x represents the spatial coordinates. The framework presented in this study is focused on steady-state problems, where the solution u does not evolve in time.

The parameterized PDE problem is to find a solution u∈S for each given parameter instance λ∈Λ such that:


(1) $$F_{x,\lambda }(u)=f_{x,\lambda },\;\forall \;x\in \Omega$$

(2) $$B_{x,\lambda }(u)=b_{x,\lambda },\;\forall \;x\in \partial \Omega ,$$

where the parameter vector λ drives the solution by affecting the operators themselves as well as the source terms here defined as $f_{x,\lambda }$ and $b_{x,\lambda }$ and S is an appropriate functional space. While the formulation above defines the problem for individual parameter values, our primary goal is to learn the *solution map*, G:Λ→S. This map takes any given parameter vector λ from the parameter space and returns the corresponding solution u=G(λ). We assume here that for any given λ in the considered parameter space, the problem is well-posed and admits a unique solution.

We approximate such solution map upon relying on a single, unified neural network, N, parameterized by trainable weights and biases θ. This network acts as a differentiable solver by learning a function that maps jointly spatial coordinates and parameter values to the solution field:


(3) $$\hat{u}(x,\lambda ;\theta )=N(x,\lambda ;\theta ).$$

Effectively, the network N represents an approximation $G_{\theta }\approx G$ of the solution map. The training process aims at discovering the optimal network parameter vector $\theta^{*}$ minimizing the residuals of the governing equations, which are scalar functions defined as:


(4) $$r_{\mathrm{pde}}(x,\lambda ;\theta )\;:\;=F[\hat{u}(x,\lambda ;\theta )]-f(x,\lambda ),$$

(5) $$r_{\mathrm{bc}}(x,\lambda ;\theta )\;:\;=B[\hat{u}(x,\lambda ;\theta )]-b(x,\lambda ).$$

The composite loss function J(θ), which is employed to constrain θ, aggregates the mean squared residuals associated with sets of collocation points. Let $T_{\mathrm{pde}}=\{(x_{i},\lambda_{i}){\}}_{i=1}^{N_{\mathrm{pde}}}$ be a set of $N_{\mathrm{pde}}$ collocation points sampled from the domain space Ω×Λ, and $T_{\mathrm{bc}}=\{(x_{j},\lambda_{j}){\}}_{\;j=1}^{N_{\mathrm{bc}}}$ be a set of $N_{\mathrm{bc}}$ collocation points from the boundary space ∂Ω×Λ. The loss function is then:


(6) $$J(\theta )=\frac{1}{N_{\mathrm{pde}}}\sum_{i=1}^{N_{\mathrm{pde}}}{\left(r_{\mathrm{pde}}(x_{i},\lambda_{i};\theta )\right)}^{2}+\frac{1}{N_{\mathrm{bc}}}\sum_{\;j=1}^{N_{\mathrm{bc}}}{\left(r_{\mathrm{bc}}(x_{j},\lambda_{j};\theta )\right)}^{2}.$$

Starting from these definitions, we introduce in the following the specific problem formulation adopted to solve the considered physical problem, corresponding to fluid flow across a saturated heterogeneous porous medium.

### Problem formulation: the parameterized Darcy system

We consider the problem of steady-state, single-phase fluid flow in a 2D porous medium. The setup is designed to be representative of a laboratory-scale experiment and the problem is formulated and solved on a dimensionless unit square domain, Ω=[0,1]×[0,1]. All quantities presented are therefore dimensionless, though they can be scaled to a consistent set of physical units for a specific application.

The system is governed by the continuity equation (ie mass conservation) and Darcy’s law (33). The domain boundary ∂Ω is partitioned into Dirichlet ($\Gamma_{D}$) and Neumann ($\Gamma_{N}$) portions. Our primary goal is to find the solution pair u(x)=(h(x),v(x)), consisting of dimensionless hydraulic head and the fluid velocity vector. The solution depends on a parameter vector λ∈Λ that defines the spatially heterogeneous hydraulic conductivity, K(x;λ). We consider here an isotropic porous medium. Hence, hydraulic conductivity can be described as a scalar quantity.

The governing equations, often referred to as the strong form of the PDE system, are given for a specific λ as:


(7a ) ∇⋅v(x;λ)+f(x)=0,∀x∈Ω

(7b ) v(x;λ)+K(x;λ)∇h(x;λ)=0,∀x∈Ω

(7c ) $$h(x;\lambda )=g_{D}(x),\;\forall \;x\in \Gamma_{D}$$

(7d ) $$v(x;\lambda )\cdot n=g_{N}(x),\;\forall \;x\in \Gamma_{N},$$

where f(x) is a source term (considered to be zero in this study), and n is a (outward-pointing) unit vector normal to the domain boundary. Functions $g_{D}$ and $g_{N}$ represent prescribed boundary values. Our particular set of boundary and initial conditions is inspired by the experimental work in Ref. (34). For our specific setup:

- The Dirichlet boundary, $\Gamma_{D}=\{x\in \partial \Omega \;|\;x=1,0.8<y<1\}$, represents an outlet with a zero-head condition, such that $g_{D}=0$ m.
- The Neumann boundary ($\Gamma_{N}=\partial \Omega \setminus \Gamma_{D}$) is further composed of:
  - An inlet section at $\Gamma_{N,\text{in}}=\{x\in \partial \Omega \;|\;y=0,0<x<0.2\}$, where the influx is prescribed by a parabolic profile with a peak velocity of $q_{\max}=1.0$:
    (8 ) $$g_{N}(x,y)=q_{\max}\cdot 4\left(\frac{x}{0.2}\right)\left(1-\frac{x}{0.2}\right).$$
  - Impermeable walls, $\Gamma_{N,\text{wall}}=\Gamma_{N}\setminus \Gamma_{N,\text{in}}$, where the normal flux is zero ($g_{N}=0$).

The main objective of the work is to learn an approximation of the solution operator (G:Λ→V) that maps any instance of parameter vector λ to its associated unique solution u upon satisfying system (7).

### The physics-informed learning framework

**Figure 1:**
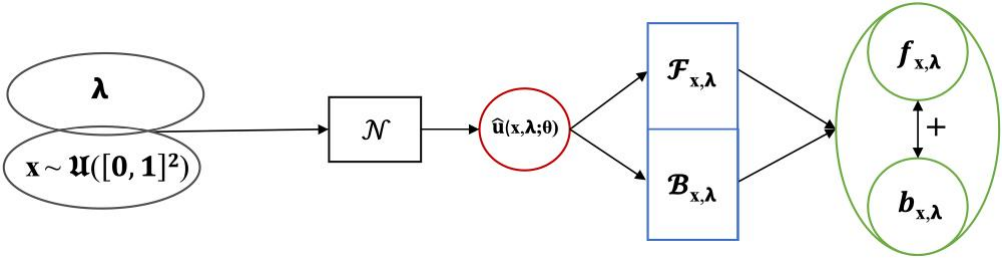
Schematic depiction of the parameterized PINN framework serving as a differentiable solver. The network N takes both spatial coordinates x and a generic instance of parameter vector λ as input to approximate the solution field $\hat{u}(x,\lambda ;\theta )$. This architecture forms the basis for learning the entire solution manifold.

Design and formulation of a PINN functioning as a *differentiable solver* are at the core of our methodology. As illustrated in Fig. 1, we use a single neural network that jointly processes both spatial and parameter inputs. Such network learns to map from the combined spatial domain and parameter space directly onto the solution manifold. The network takes space coordinates x and a parameter vector λ as inputs and renders the corresponding solution field (u) as its output. This architecture enables a single trained model to represent the entire family of solutions across the parameter space *Λ*.

#### The neural solver ansatz

We approximate the solution operator by learning the scalar hydraulic head field directly. A single, unified neural network, N, parameterized by trainable weights and biases (forming the entries of vector θ), is defined to map spatial coordinates and parameters to head values corresponding to network outputs:


(9 ) $$\hat{h}(x,\lambda ;\theta )=N(x,\lambda ;\theta ).$$

The ensuing velocity field, $\hat{v}$, is not a direct output of the network. Instead, it is obtained from the head output by applying AD (35) to enforce momentum balance, as embedded in Darcy’s law (Eq. 7b):


(10 ) $$\hat{v}(x;\lambda )=-K(x;\lambda )\nabla \hat{h}(x;\lambda ).$$

This formulation ensures that the learned velocity field is, by construction, divergence-free with respect to the learned head field, the latter being a key aspect of the considered physical problem. We employ a network architecture inspired by PirateNets (32), which is here selected for its stability (see Supplementary material, Section S1).

#### The physics-informed loss function

Values of network parameters θ are optimized by minimizing a composite loss function (denoted as J(θ)) constructed from the residuals of the governing physical laws. This loss function comprises distinct terms for the PDE residual within the domain and for each type of boundary condition. The core principle is that these physical laws must be satisfied across the whole spatial domain x∈Ω and for the whole parameter space λ∈Λ. Hence, we define the joint spatio-parameter domain as $\Omega_{\lambda }=\Omega \times \Lambda$.

##### Domain residuals (with respect to mass conservation and Darcy’s law)

Residuals enforce the governing physical laws throughout the domain $\Omega_{\lambda }$, which includes both the spatial and parametric domains. These are defined as:


(11 ) $$r_{\mathrm{cont}}(x,\lambda ;\theta )\;:\;=\nabla \cdot \hat{v}(x;\lambda )+f(x),\;\forall \;(x,\lambda )\in \Omega_{\lambda }$$

##### Boundary residuals

Boundary residuals enforce the specified conditions on the domain boundary, $\partial \Omega_{\lambda }$. We define distinct residual functions for Dirichlet and Neumann conditions. These may then be applied to diverse parts of the boundary, $\partial \Omega_{\lambda ,D}$ and $\partial \Omega_{\lambda ,N}$, respectively.

Dirichlet residual ($r_{D}$): For a prescribed head condition $h(x)=g_{D}(x)$ on $\partial \Omega_{D}$, the residual measures the difference between the network output and the target value:
  (12 ) $$r_{D}(x,\lambda ;\theta )\;:\;=\hat{h}(x;\lambda )-g_{D}(x),\;\forall (x,\lambda )\in \partial \Omega_{\lambda ,D}$$
Neumann residual ($r_{N}$): For a prescribed flux condition on $\partial \Omega_{N}$, the residual measures the difference between the component of flux normal to the boundary and its target counterpart, $g_{N}(x)$. The flux is computed via AD:
  (13 ) $$r_{N}(x,\lambda ;\theta )\;:\;=\hat{v}(x;\lambda )\cdot n(x)-g_{N}(x),\;\forall \;(x,\lambda )\in \Omega_{\lambda ,N}$$
  where n(x) is the (outward) unit vector normal to the boundary.

##### Composite loss function

Following (6), the total loss function J(θ) is the weighted sum of the mean squared residuals described above. In practice, residuals (or errors) are approximated by sampling $N_{r}$ collocation points from the domain Ω×Λ, and $N_{bD}$ and $N_{bN}$ points from the Dirichlet and Neumann boundary segments, respectively. The individual loss components are:


(14 ) $$J_{\mathrm{domain}}=\frac{1}{N_{r}}\sum_{i=1}^{N_{r}}(r_{\mathrm{cont}}(x_{i},\lambda_{i};\theta ){)}^{2},$$

(15 ) $$J_{boundary}=\frac{1}{N_{bD}}\sum_{\;j=1}^{N_{bD}}(r_{D}(x_{j},\lambda_{j};\theta ){)}^{2}+\frac{1}{N_{bN}}\sum_{k=1}^{N_{bN}}(r_{N}(x_{k},\lambda_{k};\theta ){)}^{2}.$$

These components are then combined to form the total loss:


(16 ) $$J(\theta )=w_{d}J_{\mathrm{domain}}+w_{b}J_{\mathrm{boundary}}.$$

The loss weights, $w_{d}$ and $w_{b}$, are handled dynamically during optimization to ensure stable training, as described in the following.

#### Gradient balancing for stable training

A known pathology in training PINNs is the difficulty in balancing contributions of different loss components (36). A large gradient from one term can overwhelm the others, leading to poor convergence. To address this issue, we employ “Bounded GradNorm,” an adaptive gradient balancing scheme inspired by GradNorm (37).

At each training step *t*, we compute the gradients of the individual loss components with respect to the network parameters θ:


(17 ) $$g_{\mathrm{domain}}^{t}=\nabla_{\theta }J_{\mathrm{domain}}\;\text{and}\;g_{\mathrm{boundary}}^{t}=\nabla_{\theta }J_{\mathrm{boundary}}.$$

We then compute the L2 norm of these gradient vectors. The ratio of these norms at the current training step, $r_{t}=\|g_{\mathrm{boundary}}^{t}{\|}_{2}/(\|g_{\mathrm{domain}}^{t}{\|}_{2}+\epsilon )$, where ϵ is a small constant required for numerical stability, is used to update an exponential moving average (EMA) of the ratio, denoted as $\zeta_{t}$:


(18 ) $$\zeta_{t}=(1-\beta )\zeta_{t-1}+\beta r_{t}.$$

Here, $\zeta_{0}$ is initialized to 1.0 and β∈(0,1) is a weighting factor (β=0.1 in our implementation). To ensure stability, this moving average is clipped to a predefined range $\left[\zeta_{\min},\zeta_{\max}\right]$, yielding a bounded ratio $\zeta_{t}^{\mathrm{clipped}}$.

From this bounded ratio, we define the final scaling factors for the domain and boundary gradients (denoted as $\beta_{d}$ and $\beta_{b}$, respectively) as follows:


(19 ) $$\beta_{d}=\max(\zeta_{t}^{\mathrm{clipped}},1.0)\;\text{and}\;\beta_{b}=\max(1.0/\zeta_{t}^{\mathrm{clipped}},1.0).$$

This formulation ensures that one scaling factor is always 1.0 while the magnitude of the other is adjusted on the basis of the largest gradient, thus effectively maintaining a balance between their influences. The final parameter update is then performed using these scaled gradients:


(20 ) $$\Delta \theta^{t}=-\eta \left(\beta_{d}g_{\mathrm{domain}}^{t}+\beta_{b}g_{\mathrm{boundary}}^{t}\right),$$

where *η* is the learning rate.

By dynamically rescaling the gradients, this method prevents any single loss component from dominating the optimization process. This ensures that the network learns while satisfying all physical constraints concurrently, avoiding cases where the solution is physically inconsistent (eg, satisfying the boundary conditions perfectly while violating the governing PDE within the domain). This leads to a more stable and effective navigation of the complex optimization landscape.

### Differentiable parameterization of spatial heterogeneity

Our framework is based on a differentiable mapping from a low-dimensional parameter vector λ to the spatially heterogeneous hydraulic conductivity, represented by the function K(x;λ). This mapping must be differentiable with respect to two distinct inputs:

- The spatial coordinates x, a feature which is key for computing the PDE residual within the physics-informed loss function.
- The parameters λ, thus turning the trained model into a fully differentiable solver. This property is important for enabling advanced, gradient-based applications such as sensitivity analysis and inverse modeling.

We adapt this general framework to two distinct scenarios, leading to two diverse approximated strategies, as outlined in the following.

#### Scenario 1: functional parameterization via a Gaussian anomaly

In this scenario, we define *K* using a simple analytical function where λ controls the geometric properties of a spatial feature. Adoption of this strategy is suitable for scenarios where the nature of the system heterogeneity can be described by a known functional form. In this context, we analyze a system characterized by a constant background conductivity, $K_{\min}$, to which we superimpose a spatial anomaly of circular shape featuring a variation of the value of *K* and characterized by a maximum conductivity value, $K_{\max}$. The parameter vector $\lambda =(\lambda_{1},\lambda_{2})$ defines the (x,y) coordinates of the center of this anomaly, allowing it to be placed anywhere within the domain *Ω*. The spatial distribution of K(x follow an isotropic Gaussian shape centered around the location $\left(\lambda_{1},\lambda_{2}\right)$. The full conductivity field is given by:


(21) $$K(x=(x,y);\lambda )=K_{\min}+(K_{\max}-K_{\min})\exp\;\left(-\frac{(x-\lambda_{1}{)}^{2}+(y-\lambda_{2}{)}^{2}}{2\sigma_{K}^{2}}\right),$$

where $\sigma_{K}$ is a fixed hyperparameter controlling the spatial extent (width) of the feature. For the numerical analyses performed in this study, the hydraulic conductivity fields are bounded between two fixed values $K_{\min}=\exp\;(-1.5)$ and $K_{\max}=\exp\;(1.5)$.

The function defined in Eq. 21 is differentiable with respect to both x and λ, making it straightforward to embed into the computation of the physics-informed loss. In this example the parameter space *Λ* is a subset of the spatial domain *Ω*, ie it identifies the region where the high-conductivity anomaly can be found.

#### Scenario 2: AE-based latent space parameterization

**Figure 2:**
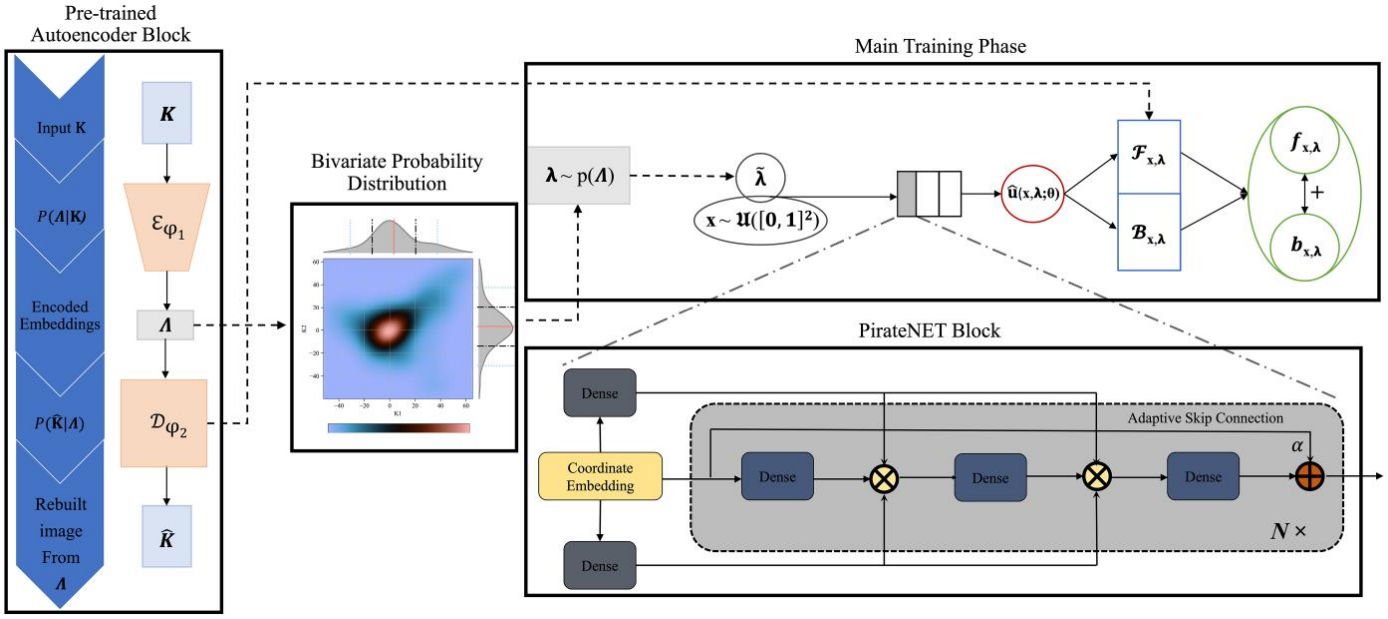
Workflow of the parameterized PINN for scenario 2. (Left) AE architecture: a CNN encoder maps reference realizations of *K* to a 2D latent vector λ. (Center) Learned 2D latent space distribution p(λ), which defines the sampling space for λ) for PINN training. (Top-right) Parameterized PINN solver, which takes spatial coordinates and latent parameters as input. The differentiable INR decoder $D_{\phi_{2}}$ is embedded into the computation of the physics-informed loss. (Bottom-right) Sketch of the PirateNet architecture (see Supplementary material, 1).

To generalize the approach to settings where a functional representation of the heterogeneity is not available, we propose a data-driven approach leveraging a pretrained AE. This corresponding workflow is illustrated in Fig. 2. It is designed to enable parameterization of a rich variety of spatially heterogeneous conductivity fields by first learning a low-dimensional latent representation of the heterogeneity and then using this representation to inform the main physics-based solver.

##### Learning a generative model of heterogeneity

The process begins by training an AE on a dataset of diverse hydraulic conductivity field realizations. These fields are generated via a selected geostatistical workflow, starting from an underlying random field. The specific statistical properties of the reference fields are detailed in the Validation of the AE-based parameterization section and the full generation algorithm is provided in Supplementary material, 2. As shown in Fig. 2 (pretrained AE block), the AE consists of a convolutional neural network (CNN) encoder ($E_{\phi_{1}}$) that maps each spatial conductivity field to a low-dimensional latent vector, $\lambda \in R^{d_{\lambda }}$ (where we use $d_{\lambda }=2$), and a INR decoder ($D_{\phi_{2}}$) that reconstructs conductivity values $\hat{K}$ at any spatial coordinate x.

Once trained on a sample of reference conductivity fields, the collection of encoded vectors λ forms an empirical multivariate distribution, denoted as p(λ). Since our latent space is 2D ($d_{\lambda }=2$), the latter is a bivariate probability distribution (as depicted in Fig. 2). This distribution defines the sampling space *Λ* that is employed during the training of the main PINN solver. Further architectural and training details for the AE are provided in Supplementary material, 2.

##### Integration of the differentiable INR decoder into the PINN

In the main training phase (Fig. 2; main training phase), the pretrained INR decoder $D_{\phi_{2}}$ is frozen and integrated directly into the PINN’s computational graph. The conductivity field is now defined as $K(x;\lambda )\;:\;=D_{\phi_{2}}(x,\lambda )$. A distinctive feature of our approach is that we leverage the full differentiability of the decoder network. Both the field value *K* and its spatial derivatives ∇K, which are needed to compute the physics loss, are calculated on-the-fly by applying AD through the decoder. This integration enables the PINN to learn the solution manifold across an entire sample of heterogeneous fields.

### Training via a multistage curriculum

Directly optimizing a deep neural network over a high-dimensional and potentially complex domain can present significant challenges, including slow convergence and susceptibility to poor local minima. To address these issues, we employ a multistage learning strategy. This approach is a significant extension of the transfer learning concepts successfully applied in our previous work (28) and aims at stabilizing the training process and accelerate convergence by progressively increasing the complexity of the learning task (38). The general methodology is formalized in Algorithm 1.

**Algorithm 1:** Multistage curriculum training for parameterized PINNs

**Data:** Governing PDE, network architecture N(⋅;θ), total epochs *E*, number of stages *S*
**Result:** Optimized global parameters $\theta_{\mathrm{global}}$
1 Initialize network parameters $\theta_{0}$
// Initial stage: pretraining on the mean parameter field
2 Define parameter subspace $\Lambda_{1}\leftarrow \{\bar{\lambda }\}$ Train N(⋅;θ) on domain $\Omega \times \Lambda_{1}$ for $E_{1}$ epochs to get parameters $\theta_{1}$
// Subsequent stages: fine-tuning on expanded parameter spaces
3 **for** s←2 **to** *S* **do**
4 Define expanded parameter subspace $\Lambda_{s}$ such that $\Lambda_{s}⊃\Lambda_{s-1}$
5 Initialize network with weights from $\theta_{s-1}$
6 Train N(⋅;θ) on domain $\Omega \times \Lambda_{s}$ for $E_{s}$ epochs to get parameters $\theta_{s}$
7 $\theta_{\mathrm{global}}\leftarrow \theta_{S}$ **return** $\theta_{\mathrm{global}}$

The core idea underpinning the curriculum described in Algorithm 1 is to first allow the network to learn the fundamental solution behavior in a simplified parameter setting before exposing it to the full variability of the parameter space. The efficacy of this strategy can be heuristically grasped through the lens of recent works on phase transitions in PINN training (39).

Training a PINN from a random initialization often involves a warm-up phase where the optimizer struggles with disordered gradients and heterogeneous residuals. Our multistage curriculum is designed to circumvent this inefficient search. The initial pretraining on the mean parameter field guides the network to a state that already captures the low-frequency solution components and establishes a baseline of residual homogeneity. Consequently, when fine-tuning begins on the full parameter space, the network is not starting from a random state, but from a well-conditioned initialization. By progressively expanding the parameter space, our method ensures that the optimizer can efficiently find and maintain a stable equilibrium, leading to faster convergence and a more robust final solution.

- **Stage 1: Pretraining on a mean or simplified field.** Training commences by restricting the parameter vector λ to a simplified representation, typically setting all parameters to their mean value, $\bar{\lambda }=E[\lambda ]$. Thus, the initial parameter subspace is $\Lambda_{1}=\{\bar{\lambda }\}$. The network N(⋅;θ) is trained on the spatial domain *Ω*. This stage enables the network to learn some average (or effective) physics of the system. Hence, it yields a robust baseline solution.
- **Subsequent stages: fine-tuning on progressively expanded parameter spaces.** After the initial pretraining, the learned network parameters $\theta_{1}$ serve as a robust and well-conditioned initialization for the next stage. In each subsequent stage *s* (from 2 to *S*), we define an expanded parameter subspace $\Lambda_{s}$ such that $\Lambda_{s-1}\subset \Lambda_{s}\subseteq \Lambda$. This expansion can be achieved according to the following strategies, depending on the nature of λ:
  - For the functional parameterization (Gaussian anomaly), if $d_{\lambda }=2$, stage 1 will fix λ to the domain center. Stage 2 releases one component (eg $\lambda_{1}$) allowing it to vary while keeping $\lambda_{2}$ fixed at its mean value. Stage 3 would then release both $\lambda_{1}$ and $\lambda_{2}$ to sample from the full 2D parameter space.
  - For the AE-based parameterization, stage 1 trains on the mean latent vector $\bar{\lambda }$ (close to 0 for a centered latent space). Stage 2 then fine-tunes the network by sampling λ from the full learned latent distribution p(λ).
  The network is then trained on the collocation points sampled from $\Omega \times \Lambda_{s}$. This process of initializing with weights from the previous (simpler) stage and fine-tuning on a more complex parameter subspace is repeated until the network is trained on the full target parameter space $\Lambda_{S}=\Lambda$.

The stage-wise training approach described above acts as a curriculum learning strategy, guiding the optimization process through a sequence of increasingly difficult tasks. By leveraging the knowledge gained in simpler settings, the network is better equipped to navigate the complex loss landscape associated with the full parameterized problem. Doing so leads to more stable training, faster convergence, and (often) more accurate and robust final solutions.

## Results and discussion

In this section, we illustrate numerical analyses designed to assess the quality of our parameterized PINN framework. We demonstrate its effectiveness and explore its physical consistency across the two distinct heterogeneity encoding strategies detailed in Methodology: differentiable physics-informed learning framework section, ie the functional parameterization via a Gaussian anomaly (Scenario 1: functional parameterization via a Gaussian anomaly section), and the more complex, data-driven approach using a pretrained AE (Scenario 2: AE-based latent space parameterization section). The problem formulation remains the same for the two test scenarios and is detailed in Problem formulation: the parameterized Darcy system section.

To quantitatively evaluate the accuracy of our parameterized PINN framework, we compare its outputs against high-fidelity reference solutions generated using a standard finite element method (FEM). The latter provides a robust and well-established numerical solution that is typically considered as baseline for solving groundwater flow settings and is detailed in Supplementary material, 3.

Results are completed by materials reported in Supplementary material. A comparison with the solution given by a PINO (25) is detailed in Supplementary material, 4 for scenario 1. The computational efficiency of our approach is discussed in Supplementary material, 5. Finally, Supplementary material, 6 documents the impact of two distinctive elements employed in our training strategy, ie curriculum learning and gradient balancing, through a dedicated ablation study. These results collectively provide quantitative support for the robustness and efficiency of the proposed approach.

Finally, Supplementary material, 7 specifies hyperparameter values employed for each scenario.

### Training dynamics and model convergence

**Figure 3:**
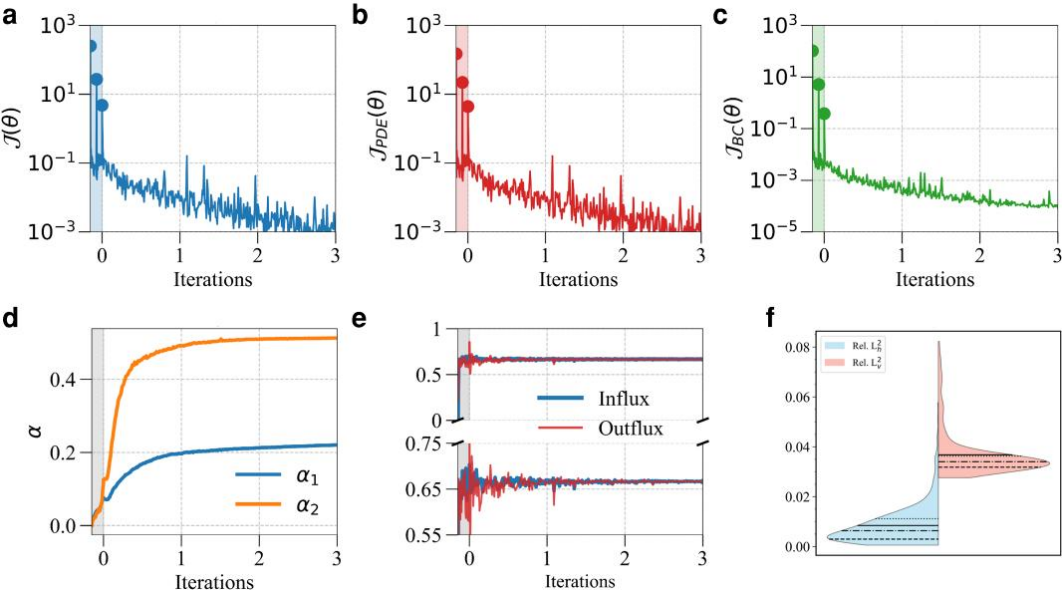
Training dynamics of scenario 1. Evolution of a) the physics-informed loss J(θ), b) the PDE residual component $J_{\mathrm{PDE}}(\theta )$, and c) the boundary condition residual component $J_{\mathrm{BC}}(\theta )$. d) Evolution of the trainable $\alpha_{i}$ (i=1,2) parameters associated with the adaptive skip connections. e) Convergence of the mean influx and outflux. Shaded regions in (a)–(e) correspond to the initial warm-up (transfer learning). Filled circles indicate the starting point for each iteration of the training phase (see Training via a multistage curriculum section). f) Violin plots showing the distribution of $\text{Rel.}\;L_{\hat{h}}^{2}$ (light blue) and $\text{Rel.}\;L_{\hat{v}}^{2}$ (salmon) errors over 1,024 parameters realizations. Mean (solid line), median (dotted line), and 25th/75th percentiles (dashed lines) are also identified.

The performance of the learned (PINN-based) surrogate models is contingent upon a stable and effective training process. Results related to the latter are depicted in Fig. 3 for the functional (scenario 1). Similar results are obtained for the AE-based scenario 2 as shown in Supplementary material, 2 (see Fig. S3).

The initial warm-up phase, where the model is trained on a single parameter instance corresponding to the center of the parameter range of variability, is highlighted by the shaded region within each subplot. Upon transitioning to training on the full parameter space, the total loss and its constituent components (Fig. 3a–c) exhibit a significant initial drop. The latter is then followed by steady convergence. This result suggest that curriculum learning is effective in leading to accurate training as further reinforced by the ablation study reported in Supplementary material, 6.1. Figure 3d displays the evolution of the trainable parameters $\alpha_{i}$ (i=1,2) within the PirateNet adaptive residual blocks (see Supplementary material, 1). These parameters are initialized at zero and their value increases during training. This trend suggests that the network progressively incorporates nonlinearities and increases its effective depth to attain improved fit of the solution manifold. Figure 3e displays the inflow and outflow evaluation, based on the PINN solution. These results confirm that our approach satisfies global mass balance with good accuracy. The violin plots (Fig. 3f) show that the majority of λ realizations are associated with low relative errors for both head and velocity. The median relative $L^{2}$ error related to model-based heads and velocity is ≈0.01 and 0.035, respectively. This supports the model ability for accurate generalization across the full solution manifold.

### Scenario 1: functional parameterization

#### Global accuracy and generalization

**Figure 4:**
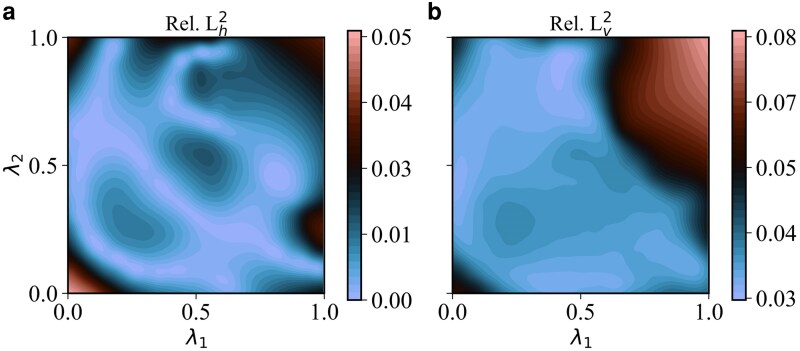
Performance evaluation for scenario 1. a) Contour plot of the relative $L^{2}$ norm error for model-based hydraulic heads ($\text{Rel.}\;L_{\hat{h}}^{2}$) across the $\lambda =(\lambda_{1},\lambda_{2})$ parameter space and b) relative $L^{2}$ norm error for the velocity field ($\text{Rel. }L_{\hat{v}}^{2}$).

Figure 4 depicts the results of a quantitative assessment of the model accuracy, evaluated over a grid of 1,024 distinct λ values and compared against reference solutions from the FEM solver. The contour plots (Fig. 4a and b) illustrate the spatial distribution of the relative $L_{2}$ error associated with the model-based hydraulic head ($\hat{h}$) and the derived velocity field ($\hat{v}$). The relative $L_{2}$ error for the velocity field is computed considering both components, thereby accounting for discrepancies in both magnitude and direction. The framework demonstrates robust generalization, with low error across the entire parameter space. Errors in the computed velocity markedly increase as the Gaussian anomaly interferes with the outflow boundary conditions (see top-right corner in Fig. 4b). This result is related to the presence of sharp velocity gradients close to the outlet section, that are challenging to approximate in the presence of local variations of *K*.

#### Local mass conservation

**Figure 5:**
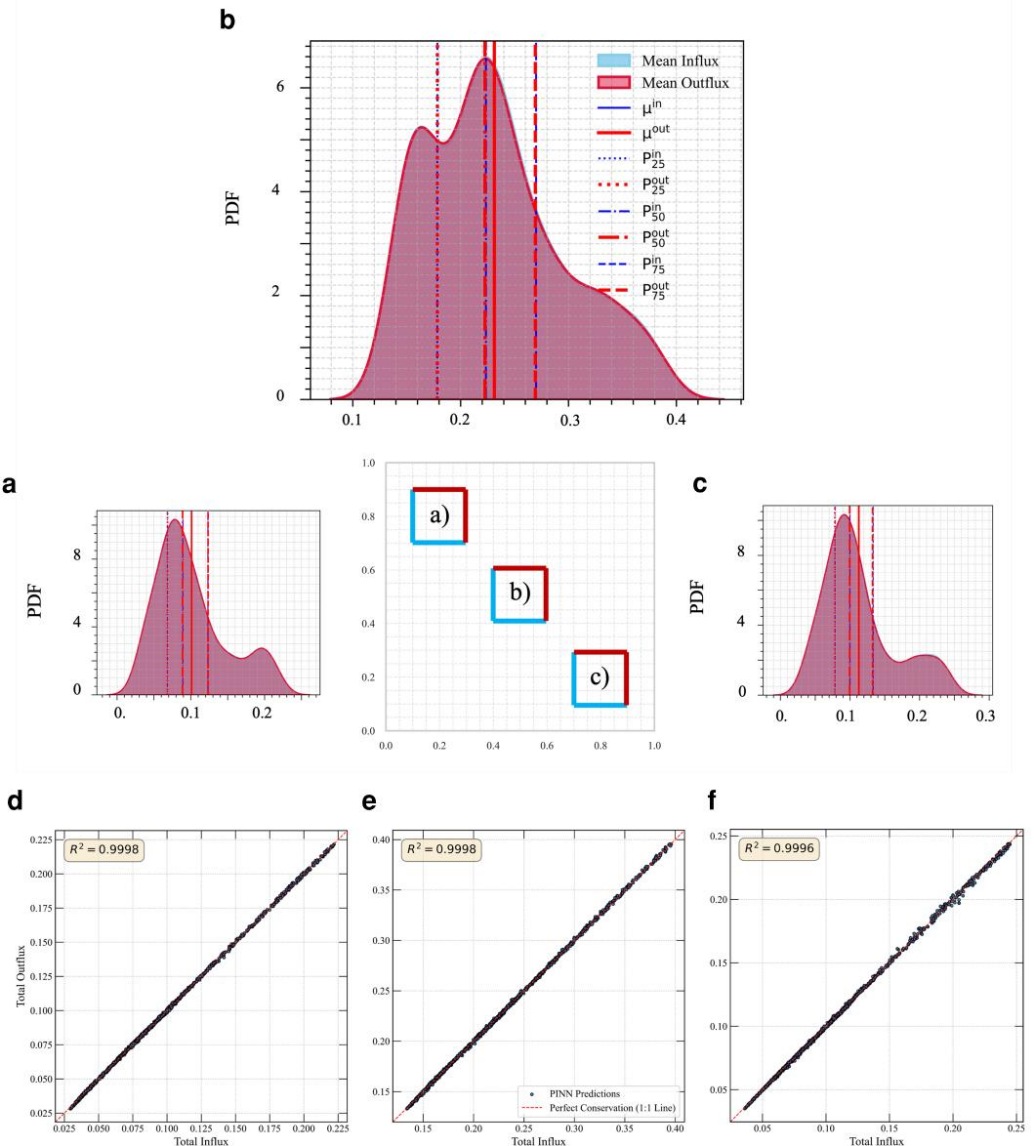
Assessment of local mass conservation for the scenario 1. Comparison of the statistics via the PDFs of total influx and outflux for the three control volumes considered (a–c), each identified in the central panel; pairwise validation of influx and outflux via scatter plots for the same three control volumes across the whole test sample (d–f), each panel refers to one control volume.

Beyond global error metrics, we investigate whether the learned solutions guarantees mass conservation in selected control volumes. We consider three selected control volumes within the domain (labeled in Fig. 5a–c).

As a first analysis (Fig. 5a–c), we compare the probability density functions (PDFs) of the total influx and outflux for each control volume across the 1,024-realization ensemble. The overlap of the distributions suggests that influx and outflux are equivalent from a statistical standpoint. We further perform a direct, pairwise comparison of fluxes (Fig. 5d–f, each referring to one volume in a–c, respectively). Each point in these scatter plots represents a single parameter realization, values of computed influx and outflux being associated with horizontal and vertical axis, respectively. These results demonstrate mass conservation. Data points align along the 1:1 line, which represents conservation, for all three control volumes. These results imbue us with confidence that the PINN, despite being trained only on the global PDE residual, successfully learns solutions that implicitly and robustly satisfy mass conservation in arbitrary control volumes on a per-realization basis.

#### Application to uncertainty quantification

**Figure 6:**
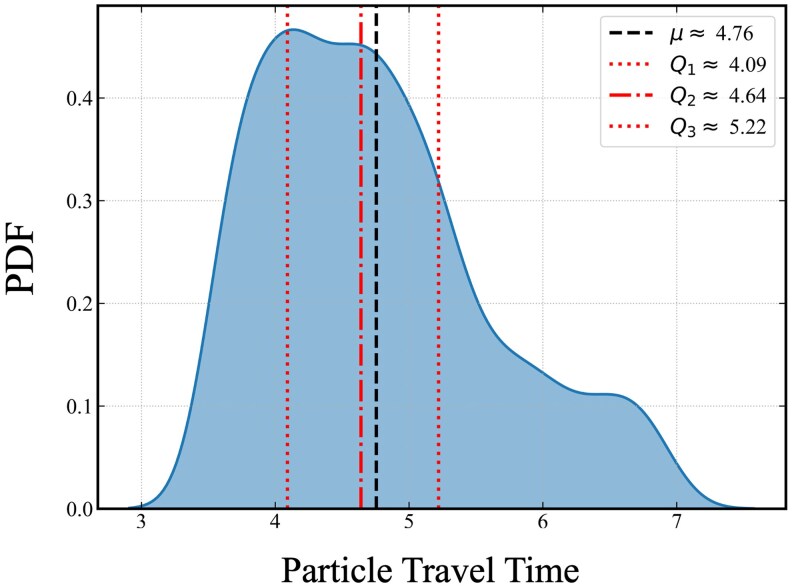
Travel time distribution for scenario 1. PDF of inlet-to-outlet travel times estimated via advective particle tracking, considering injection of a particle at location $x_{0}=0.1$ m, $y_{0}=0.001$ m). The sample mean (dashed line) and the quartiles $Q_{i}$ of the distribution are denoted through vertical lines (dotted lines).

To showcase a practical application of the framework, we perform a Monte Carlo analysis considering a set of particle tracking simulations to estimate the distribution of advective travel times. The results of this analysis are depicted in Fig. 6. The Monte Carlo ensemble is formed by $2^{10}=1,024$ distinct hydraulic conductivity realizations randomly generated by sampling from the parameter space *Λ*.

For each of these 1,024 realizations, we simulate the trajectory of a single particle. The latter is introduced at a fixed starting location at the domain inlet, $x_{0}=(0.1,0.001)$ and is advected across the corresponding steady-state velocity field stemming from the trained PINN surrogate. The particle movement is computed using a first-order Forward Euler integration scheme with a fixed time step of Δt=0.01.

A given simulation is terminated once the particle reaches the defined outlet region ($\Gamma_{D}$), the total elapsed time is recorded as its advective travel time. The resulting distribution of these 1,024 travel times reveals significant variability (with a mean of 4.76 and a median of 4.64). Our results display a skewed distribution, being characterized by a mild right-tail. This outcome is likely due to the slight asymmetry of the considered setup, resulting from the imposed inlet–outlet location, which in turn results in an asymmetric effect of the hydraulic conductivity perturbation.

### Scenario 2: AE-based latent space parameterization

Here, we focus on the more complex case where heterogeneity is defined by a low-dimensional latent vector λ from a pretrained AE.

#### Validation of the AE-based parameterization

To validate the AE, we must first define the reference hydraulic conductivity *K*, it was trained to reproduce. The generation process (detailed in Supplementary material, 2) starts by creating an ensemble of standard, zero-mean Gaussian random fields (GRFs) and an isotropic correlation length of ℓ=0.15. In the hydrogeological context, this underlying field is conventionally interpreted as the log-conductivity, Y=ln(K). These raw *Y* fields are then subjected to a deterministic transformation (exponentiation followed by linear scaling, see Supplementary material, 2) to produce the final training dataset with $K\in [e^{-0.8},e^{0.8}]\approx [0.45,2.22]$.

**Figure 7:**
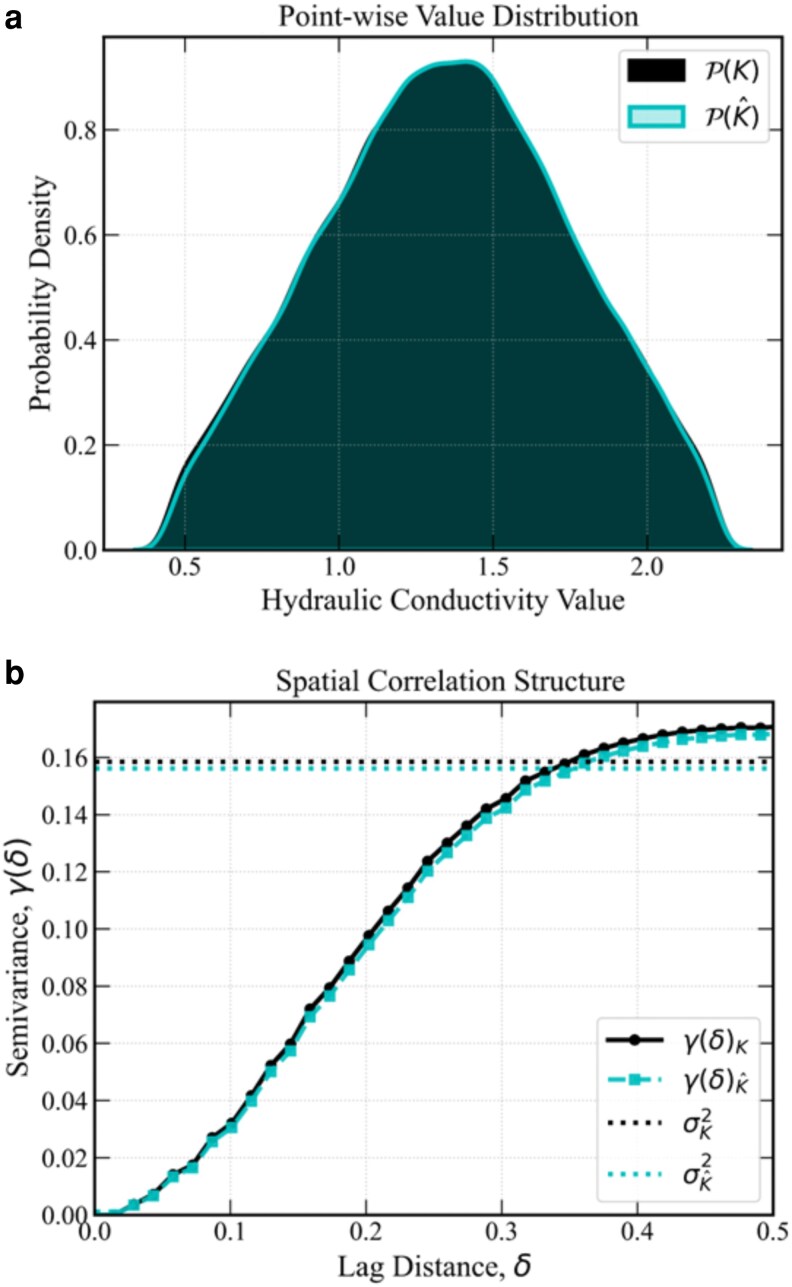
Quantitative assessment of the AE reconstruction capability. Comparison between statistical properties of an ensemble of original training *K* fields and their corresponding (model-based) reconstructions $\hat{K}$. a) PDFs of *K* and $\hat{K}$; b) comparison of empirical semivariograms.

The latter transformation defines the statistical properties of the reference ensemble used for our analysis. The fields are characterized by an ensemble mean conductivity of $\bar{K}\approx 1.35$ and a total ensemble variance of ≈0.1583 (represented by the black dotted line in Fig. 7b). We note that this total variance is the sum of the average spatial variance within realizations ≈0.146 and the variance of the mean values across realizations ≈0.0123. By definition, for a stationary field, the sill of the semivariogram (corresponding to the plateau attained at large distances where data are uncorrelated) equals the variance of the field. As observed in the spatial correlation structure, the semivariogram sill stabilizes at a slightly higher value ($\gamma_{\mathrm{sill}}\approx 0.17$). This discrepancy is a well-known statistical consequence of the finite domain size relative to the correlation length (L≈6.6ℓ). In finite domains, the sample variance tends to underestimate the true global variance of the process because it is calculated relative to the local mean of the realizations rather than the global population mean. The variogram sill, therefore, provides a more robust estimate of the total structural variability of the generated fields. Overall, this analysis provides evidence that our AE successfully preserves second-order spatial statistics, including both the field correlation length and the total spatial variance ($\sigma_{K}^{2}$).

##### Assessment of the AE performance

Visual comparison supports the capability of the AE in reproducing the expected heterogeneity features (see Supplementary material, 2, Fig. S2). We perform here a quantitative analysis grounded on the comparison between two key statistical features across an ensemble of 512 fields. Figure 7 embeds the results of this analysis. We start by comparing the point-wise PDF of the conductivity values (Fig. 7a). The overlap of the distributions for the original (P(K)) and reconstructed ($P(\hat{K})$) fields provides strong evidence that the AE accurately preserves the statistics (the correct range and frequency distribution of conductivity values) of the reference field. We then compare the spatial correlation structure by evaluating the empirical semivariograms (Fig. 7b). We observe a satisfactory agreement between the semivariograms of the original ($\gamma (\delta {)}_{K}$) and reconstructed ($\gamma (\delta {)}_{\hat{K}}$) ensembles.

Note that the quality of the performance evidenced here is related to the specific characteristics of the considered reference fields. A comprehensive analysis across heterogeneous fields displaying a wide range of statistical properties and correlation structures is beyond the scope of this study.

##### Performance on individual realizations

**Figure 8:**
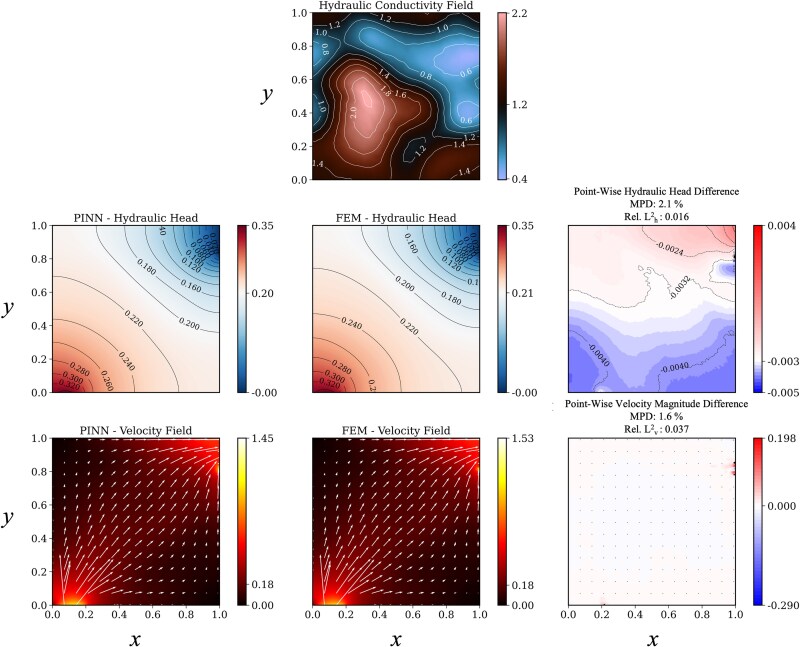
Scenario 2 best case scenario among 512 test realizations (lowest relative $L^{2}$ error in velocity). (Top row) The specific heterogeneous hydraulic conductivity $K(x;\lambda_{\mathrm{best}})$ generated by the INR decoder for the selected latent vector $\lambda_{\mathrm{best}})$. (Middle row, from left to right) Hydraulic head field resulting from the PINN ($\hat{h}$); reference hydraulic head field obtained through the FEM solver ($h_{\mathrm{FEM}}$); point-wise difference ($h_{\mathrm{FEM}}-\hat{h}$) between FEM and PINN head solutions (MPD and relative $L^{2}$ norm error are also indicated). (Bottom row, from left to right) Velocity field (magnitude contours and quiver plot) ($\hat{v}$); reference velocity field from FEM solver ($v_{\mathrm{FEM}}$); point-wise difference in velocity magnitude ($\left\|v_{\mathrm{FEM}}\|-\|\hat{v}\right\|$) between FEM- and PINN-based solutions, including MPD and relative $L^{2}$ norm error for velocity magnitude.

**Figure 9:**
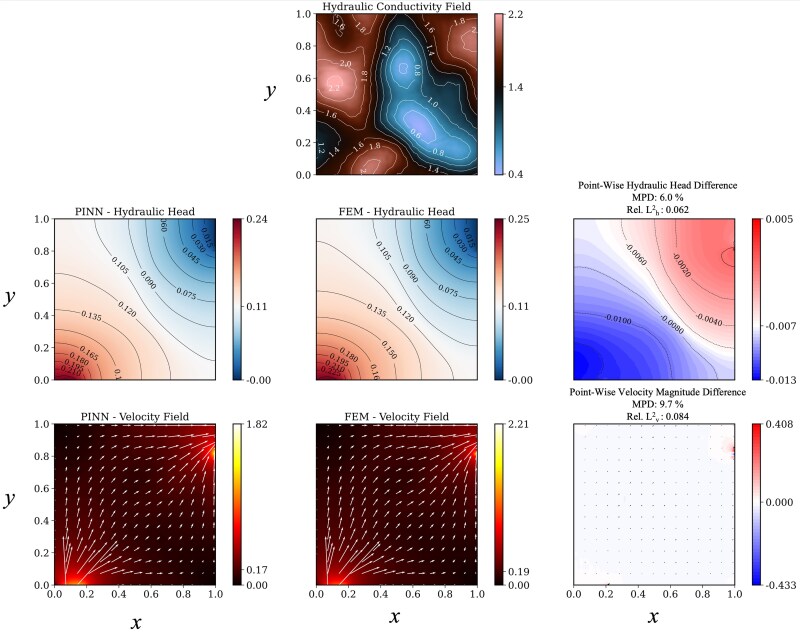
Scenario 2 best case scenario among 512 test realizations (highest relative $L^{2}$ error in velocity). (Top row) The specific heterogeneous hydraulic conductivity field $K(x;\lambda_{\mathrm{worst}})$ generated by the INR decoder for the selected latent vector $\lambda_{\mathrm{worst}}$. (Middle row, from left to right) Hydraulic head field evaluated through the PINN ($\hat{h}$); reference hydraulic head field based on the FEM solver ($h_{\mathrm{FEM}}$); point-wise difference ($h_{\mathrm{FEM}}-\hat{h}$) between FEM and PINN head solutions (MPD and relative $L^{2}$ norm error are also indicated). (Bottom Row, from left to right) Velocity field (magnitude contours and quiver plot) derived from the PINN’s head output via AD ($\hat{v}$); reference velocity field from FEM solver ($v_{\mathrm{FEM}}$); point-wise difference in velocity magnitude ($\left\|v_{\mathrm{FEM}}\|-\|\hat{v}\right\|$) between FEM- and PINN-based solutions, including MPD and relative $L^{2}$ norm error for velocity magnitude.

To illustrate the performance in specific instances, Figs. 8 and 9 depict exemplary results corresponding to the best- ($K(x;\lambda_{\mathrm{best}})$) and worst-case ($K(x;\lambda_{\mathrm{worst}})$) scenarios (in terms of velocity error, evaluated against the FEM reference solution). Results are evaluated from a sample of 512 Monte Carlo realizations. In the best-case setting (Fig. 8), PINN results for both head and velocity display excellent agreement with their FEM reference counterparts, with a mean percentage difference (MPD) of 2.1% for head and 1.6% for velocity. In the worst-case scenario (Fig. 9), the PINN still maintains a good qualitative agreement with the reference solution, with an MPD of 6.0% for head and 9.7% for velocity. Notably, this particular realization is characterized by the presence of a high conductivity region close to the outlet. This feature could underpin the relatively large errors obtained across that particular region. While representing an outlier, this setting helps to define the performance envelope of the model and highlights its robustness even for complex conductivity structures.

##### Analysis of flow characteristics

**Figure 10:**
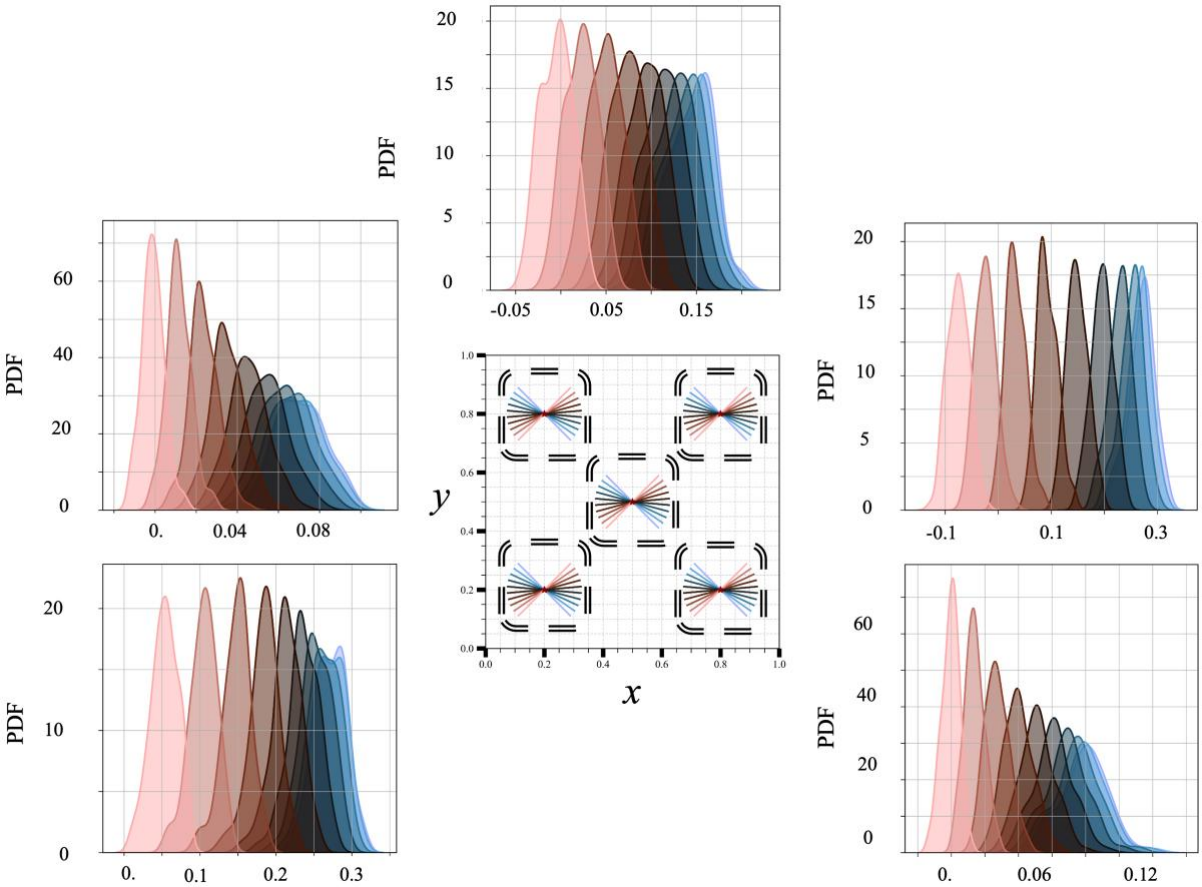
Directional flux variability for scenario 2. Central panel: Target locations and selected line segments (0.2) oriented at different angles (eg increments of $22.5^{\circ }$ from $0^{\circ }$ to $157.5^{\circ }$, totaling eight orientations). Side panels: Sample PDF of the net flux across all 512 test realizations of λ. Each PDF is color-coded according to the corresponding line angle.

To explore the directional characteristics and inherent uncertainty of the flow field predicted by our parameterized PINN, we analyze the flux across line segments at varying orientations. Figure 10 presents this analysis for the AE-based case. At five distinct central points within the domain, a set of co-located short line segments (length 0.2) were defined, each rotated to a different orientation.

For each of the 512 conductivity realizations, the net flux through each oriented line segment is calculated by integrating the PINN-derived velocity component normal to the line. The resulting sample PDFs of these directional fluxes are displayed in the surrounding subplots, each associated with its corresponding line orientation and central position. The observed variations in the shape, spread, and central tendency (mean or modal value) of these PDFs across different angles and spatial locations underlines the anisotropic nature of the flux uncertainty. For instance, at a given location, some orientations yield narrowly distributed flux values, while others exhibit broader variability, reflecting the combined effects of heterogeneity and boundary conditions. Our approach enables one to efficiently perform this type of analysis. The results of the latter reveal preferential flow directions and flux sensitivity to conductivity variations. We recall that these results would be computationally expensive to obtain via conventional Monte Carlo simulations, especially when evaluating fluxes along arbitrary lines across the domain. The PINN ability to provide continuous, differentiable solution fields allows for such flexible postprocessing and interrogation of the flow field.

Finally, we characterize the spatial distribution of flow uncertainty. We recall (see Validation of the AE-based parameterization section) that the input heterogeneity is driven by an underlying conductivity field, *K*, with a variance of $\sigma_{K}^{2}\approx 0.1583$. This value, representing the *input uncertainty*, corresponds to a moderate level of heterogeneity, characteristic of many sedimentary aquifer systems. In the following, we analyze the resulting output uncertainty upon relying on the local value of the coefficient of variation (CV=σ/μ) of the velocity norm.

**Figure 11:**
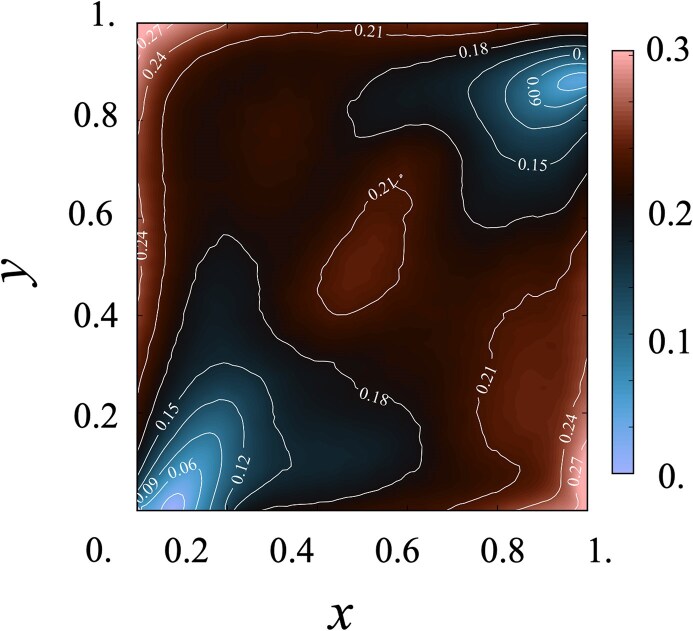
Scenario 2: spatial map of relative uncertainty associated with the flow field. Coefficient of variation (CV) of the velocity norm, evaluated across an ensemble of 512 conductivity realizations.

Figure 11 presents the spatial map of CV values of velocity norms. Near the inlet and outlet, the flow is strongly constrained by the prescribed boundary conditions, leading to low velocity variability (and thus low CV), as observed in the plot. In contrast, the flow paths in the interior of the domain are highly sensitive to the specific realization of the heterogeneous conductivity field. It is in these regions, where the flow must navigate the complex conductivity field, that the velocity exhibits the highest variability relative to its mean, leading to the high CV values. This quantitative view of relative uncertainty, which highlights the areas of greatest predictive uncertainty away from the boundaries, offers comprehensive insights into the spatial structure of the flow field statistics.

## Conclusions and future perspectives

This study introduces a general and scalable framework recasting a PINN as a differentiable physics solver. The latter is exemplified through the solution of steady-state Darcy flow taking place in heterogeneous porous media. As a distinctive feature, the proposed approach captures the effects of parameter variability, thereby addressing uncertainty arising from incomplete knowledge of subsurface properties. By training a single, unified network on physics-based residuals over a joint spatial and parametric domain, the model learns an entire manifold of physically consistent solutions. By allowing accurate and fast differentiability of the solution with respect to spatial and parametric inputs our method can provide benefits across a broad range of applications, both in forward and inverse modeling efforts. Key findings and contributions can be summarized as follows:

- Our modeling framework enables direct solution of the physical problem across a defined parameter space, effectively extending the PINN-UU concept (28) to spatially heterogeneous parameter fields. We demonstrate that a single neural network can learn to approximate heterogeneous Darcy flow solutions across a continuous range of governing parameters.
- We introduce and validate two distinct strategies for the differentiable parameterization of spatial heterogeneity of model parameters: (i) a direct functional approach for simple geometric features and (ii) a data-driven approach using a pretrained, capable of handling parameters spatial distributions through a coordinate-based AE.
- A key technical innovation of the study is the direct integration of the AE’s differentiable decoder based on an implicit neural representation (INR) into the PINN loss function. This enables on-the-fly computation of the conductivity field and its spatial derivatives via AD, which constitutes a critical step for enforcing the physics-based PDE residual.
- Through an extensive set of numerical analyses, we demonstrate that the learned solutions are robust and consistent with physical principles, such as local mass conservation, even as training is performed solely on the global PDE residuals. This highlights the ability of our framework to internalize the underlying physics.
- Computational efficiency of the trained model is documented to enable practical uncertainty quantification (UQ) tasks, such as those related to simulation of transport upon relying on a large-ensemble of particle tracking evaluations.

Our work points toward several future directions. These include, for example, extending this methodology to transient flow and transport scenarios. Further investigations could address more advanced generative models for encoding more complex geological structures. For example, moving beyond the current INR-decoder to consider variational or generative adversarial approaches constitutes an interesting avenue for future research. Finally, trained solution operator can serve as a robust prior that can be rapidly fine-tuned or conditioned on sparse, possibly noisy, experimental observations using (stochastic) inverse modeling techniques.

## Supplementary Material

pgag195_Supplementary_Data

## Data Availability

Codes used in this article will be available in the following github repository https://github.com/MiladPnh/DarcyPINN-CausalTraining.git.

## Preprints

This manuscript was posted on a preprint: https://doi.org/10.48550/arXiv.2601.16350.
