# Supplementary material for: Physics-informed differentiable solvers for learning parametric solution manifolds in heterogeneous physical systems
Source: PNAS Nexus. 2026 Jun 1;5(6):pgag195. doi: 10.1093/pnasnexus/pgag195 (PMC13275013; doi:10.1093/pnasnexus/pgag195)
Supplement: pgag195_Supplementary_Data [file pgag195_supplementary_data.pdf]

PNAS Nexus, 2025, pp. 1–14

doi:

Supporting Information: Physics Informed Differentiable Solvers for Learning Parametric Solution Manifolds in Heterogeneous Physical Systems

Milad Panahi,[a] Giovanni Michele Porta,[a, *] Monica Riva[a] and Alberto Guadagnini[a]

[a]Dipartimento di Ingegneria Civile e Ambientale, Politecnico di Milano, Piazza L. da Vinci 32, Milano, 20133, Italy
[*]To whom correspondence should be addressed: giovanni.porta@polimi.it

Abstract

This file provides supporting information for the paper entitled "Physics Informed Differentiable Solvers for Learning Parametric Solution Manifolds in Heterogeneous Physical Systems", submitted to PNAS Nexus. Table of contents:
Section 1: PirateNet Architecture Details.
Section 2: Autoencoder Data Generation and Architecture
Section 3: FEM as baseline
Section 4: Comparison with Physics-Informed Neural Operators (PINO)
Section 5: Computational Efficiency, Amortized Cost, and Scaling Behavior
Section 6: Ablation Study: Architecture and Optimization Strategies
Section 7: Hyperparameter Configurations

Supporting information

1. PirateNet Architecture Details

The core of our modeling framework is a parameterized Physics-Informed Neural Network (PINN) whose architecture, depicted in Figure S1, is inspired by the PirateNet design Wang et al. [2024]. This design was chosen for its demonstrated ability to facilitate stable and efficient training of deep neural networks in the context of physics-informed learning. The architecture can be broken down into three main components: an input embedding layer, a series of adaptive residual blocks, and a final output layer.

The network is structured to accept both spatial coordinates $\mathbf{x}$ (sampled uniformly from the unit square) and a low-dimensional parameter latent vector $\boldsymbol{\lambda}$ as concatenated input. This combined input first passes through an initial dense layer for coordinate embedding, followed by processing through a series of adaptive residual blocks. As detailed in the magnified portion of Figure S1, each PirateNet block features internal dense layers, gating mechanisms based on the initial embedding, and a crucial adaptive skip connection modulated by a trainable scalar α. This architecture facilitates the learning of a "solution bundle", where the network's output $u_{\boldsymbol{\Theta}_\lambda}$, is a function of both space and the latent space parameters defining the hydraulic conductivity spatial structure. The network is trained by minimizing a loss function derived from the residuals of the Darcy flow governing equations ($\mathcal{F}_{\mathbf{x},\boldsymbol{\lambda}}$) and boundary conditions ($\mathcal{B}_{\mathbf{x},\boldsymbol{\lambda}}$), ensuring physical consistency across the entire spatio-parameter domain without reliance on pre-computed simulation data.

Input Embedding with Random Fourier Features. To mitigate the well-known spectral bias of Multilayer Perceptrons (MLPs), the normalized input coordinates $(\mathbf{x}, \boldsymbol{\lambda})$ are mapped to a higher-dimensional feature space using a Random Fourier Feature (RFF) embedding. We define the combined input vector $\mathbf{z} = (\mathbf{x}, \boldsymbol{\lambda})$, where $\mathbf{z} \in [0,1]^{d_z}$ and $d_z = d_x + d_\lambda$. The embedding is given by:

$$\Phi(\mathbf{z}) = [\cos(2\pi\mathbf{B}\mathbf{z}), \sin(2\pi\mathbf{B}\mathbf{z})]^T \,, \tag{S1}$$

where the entries of $\mathbf{B} \in \mathbb{R}^{m\times d_z}$ are sampled i.i.d. from an isotropic Gaussian distribution $\mathcal{N}(0,1)$ and fixed during training. This embedding enhances the network's ability to approximate solutions with rich spatial variability, overcoming the tendency of standard MLPs to over-smooth the output. The output of this layer, denoted $\Phi(\mathbf{z})$, is then passed to the main body of the network.

© The Authors. All rights reserved.

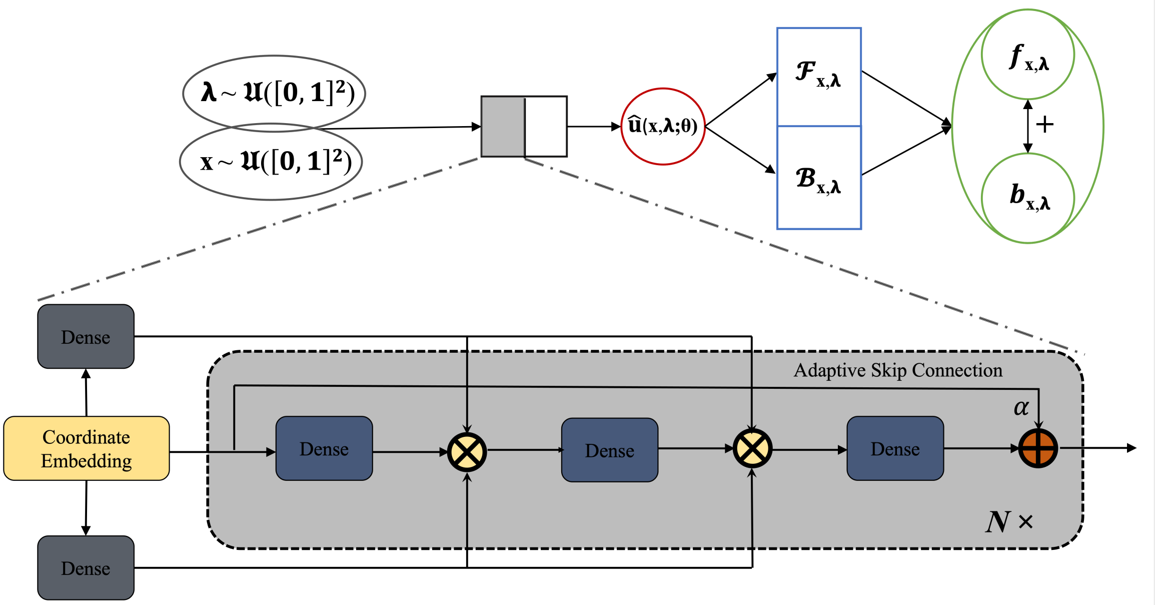

Fig. S1. Schematic of the parameterized Physics-Informed Neural Network (PINN) architecture based on PirateNet for solving the steady-state Darcy flow problem. The network takes spatial coordinates $\mathbf{x} \sim \mathcal{U}([0,1]^2)$ and parameter vectors $\boldsymbol{\lambda} \sim \mathcal{U}([0,1]^2)$ (representing, e.g., the center of a Gaussian conductivity feature) as input. The input undergoes coordinate embedding before being processed by two adaptive residual blocks ($N\times$, where N=2 in this study), detailed in the zoomed-in view. The output u_{Θ_λ} is then used to evaluate the physics-informed loss, which includes residuals of the governing PDE operator $\mathcal{F}_{\mathbf{x},\boldsymbol{\lambda}}$ and boundary condition operator $\mathcal{B}_{\mathbf{x},\boldsymbol{\lambda}}$, compared against source terms $f_{\mathbf{x},\boldsymbol{\lambda}}$ and boundary values $b_{\mathbf{x},\boldsymbol{\lambda}}$, respectively. The adaptive skip connection within each PirateNet block is controlled by a trainable scalar α.

Following the embedding, we compute two global gating layers, $\mathbf{U}$ and $\mathbf{V}$, via two dense layers:

$$\mathbf{U} = \sigma(\mathbf{W}_u \Phi(\mathbf{z}) + \mathbf{b}_u), \quad \mathbf{V} = \sigma(\mathbf{W}_v \Phi(\mathbf{z}) + \mathbf{b}_v), \tag{S2}$$

where σ is the activation function. These two encoding maps act as global gates in each residual block of the architecture, that modulate the information flow based on the inputs $\mathbf{z}$ [Wang et al., 2021, Anagnostopoulos et al., 2023].

Adaptive Residual Blocks. The network comprises L residual blocks. Let $\mathbf{z}^{(l)}$ denote the input to the l-th block (for $1 \leq l \leq L$) and $\mathbf{z}^{(0)} = (\mathbf{x}, \boldsymbol{\lambda})$ as initial input. The forward pass through the block matches the formulation in [Wang et al., 2024], adapted here for our parameterized input $\mathbf{z}$. It involves three dense operations and two gating steps:

$$\mathbf{f}^{(l)} = \sigma(\mathbf{W}_1^{(l)} \mathbf{z}^{(l)} + \mathbf{b}_1^{(l)}) \tag{S3}$$

$$\mathbf{m}_1^{(l)} = \mathbf{f}^{(l)} \odot \mathbf{U} + (1 - \mathbf{f}^{(l)}) \odot \mathbf{V} \tag{S4}$$

$$\mathbf{g}^{(l)} = \sigma(\mathbf{W}_2^{(l)} \mathbf{m}_1^{(l)} + \mathbf{b}_2^{(l)}) \tag{S5}$$

$$\mathbf{m}_2^{(l)} = \mathbf{g}^{(l)} \odot \mathbf{U} + (1 - \mathbf{g}^{(l)}) \odot \mathbf{V} \tag{S6}$$

$$\mathbf{n}^{(l)} = \sigma(\mathbf{W}_3^{(l)} \mathbf{m}_2^{(l)} + \mathbf{b}_3^{(l)}) \tag{S7}$$

$$\mathbf{z}^{(l+1)} = \alpha^{(l)} \mathbf{n}^{(l)} + (1 - \alpha^{(l)}) \mathbf{z}^{(l)} \tag{S8}$$

Here, $\odot$ denotes element-wise multiplication, σ is a non-linear activation function (e.g., Tanh), and the terms $\mathbf{W}$ and $\mathbf{b}$ represent trainable weight matrices initialized by the Glorot scheme [Glorot and Bengio, 2010] and bias vectors, respectively. We introduce the symbol $\mathbf{n}^{(l)}$ (Eq. S7) to represent the block's non-linear mapping, distinguishing it from the hydraulic head h.

Final Output Layer and Initialization Properties. The output of the final residual block, $\mathbf{z}^{(L+1)}$, is projected to the physical solution variable (the hydraulic head in this work) via a final linear dense layer:

$$u_{\Theta_\lambda}(\mathbf{x}, \boldsymbol{\lambda}) = \mathbf{W}_{(L+1)} \mathbf{z}^{(L+1)} + b_{out}, \tag{S9}$$

where $\mathbf{W}_{(L+1)}$ and $b_{(L+1)}$ are the final learnable weight matrix and bias.

The key innovation lies in Eq. (S8), where $\alpha^{(l)} \in \mathbb{R}$ is a trainable scalar parameter. A defining characteristic of the PirateNet architecture is its behavior at the start of training. Since the skip-connection parameters $\alpha^{(l)}$ are initialized to zero, every residual block initially functions as an identity map (i.e., $\mathbf{z}^{(l+1)} = \mathbf{z}^{(l)}$). By induction, the input to the final layer at initialization is

identical to the output of the first embedding layer, $\mathbf{z}^{(L+1)} = \Phi(\mathbf{z})$. Consequently, at step zero, the entire deep network collapses mathematically into a linear combination of the first layer embeddings at initialization:

$$u^{\text{init}}_{\Theta_\lambda}(\mathbf{z}) = \mathbf{W}_{(L+1)}\Phi(\mathbf{z}) + b_{(L+1)}. \tag{S10}$$

This property is significant for two reasons. First, it circumvents the "initialization pathologies" often observed in deep PINNs, where random weight initialization in deep non-linear networks can lead to vanishing gradients or stiff optimization landscapes. By starting as a shallow linear model, the network ensures stable gradient flow. As training progresses and $\alpha^{(l)}$ evolves, the network progressively "unlocks" its depth and non-linearity, increasing its expressivity only as required to minimize the PDE residuals.

Second, this formulation allows the network to be viewed as a linear expansion of basis functions at initialization. This structure enables the optional integration of prior knowledge or available data (e.g., boundary conditions or sparse measurements $\mathbf{Y}$) directly into the initialization phase. One can solve for the optimal initial weights $\mathbf{W}_{out}$ by minimizing a standard least-squares objective:

$$\min_{\mathbf{W}_{out}} \|\mathbf{W}_{out}\Phi(\mathbf{z}) - \mathbf{Y}\|_2^2. \tag{S11}$$

This capability provides a mathematically robust "initial guess" for the solver, akin to spectral methods, before the physics-informed optimization begins fine-tuning the non-linear parameters.

2. Autoencoder Data Generation and Architecture

This Supporting Information provides supplementary details regarding the generation of training data and the specific architecture of the autoencoder (AE) used in Scenario 2 (see Section 2.4.2). The AE is employed to derive a low-dimensional parameterization $\boldsymbol{\lambda}$ for imposing the spatial distribution fo hydraulic conductivity $K(\mathbf{x})$, which is then used as input to the main physics-informed neural network (PINN) solver.

2.1. Gaussian Random Field (GRF) Sample Generation

The training dataset for the autoencoder consists of M realizations of hydraulic conductivity, generated as two-dimensional Random Fields. We utilize a spectral method based on the Fast Fourier Transform (FFT) for efficient generation. This method ensures the generated fields possess specified statistical properties, primarily governed by their power spectral density (PSD).

The generation process for each sample on an $N \times N$ grid (with $N = 32$ in our setup) involves the following steps:

(i) **Frequency Grid:** Define the discrete spatial frequencies (k_x, k_y) corresponding to the $N \times N$ grid using `FFT`.

(ii) **Power Spectral Density (PSD):** Define the PSD, $S(k_x, k_y)$, corresponding to a desired covariance model. For a Gaussian covariance function with correlation length $L = 0.15$, the PSD is given by:

$$S(k_x, k_y) = C \exp\left(-2(\pi L)^2(k_x^2 + k_y^2)\right) \tag{S12}$$

where C is a normalization constant.

(iii) **Random Fourier Coefficients:** Generate a realization of complex Gaussian white noise $\eta(k_x, k_y)$ in the frequency domain, where the real and imaginary parts are drawn independently from $\mathcal{N}(0, 1)$.

(iv) **Field in Fourier Domain:** Construct the Fourier representation of the GRF, $K_{FFT}(k_x, k_y)$, by scaling the noise with the square root of the PSD:

$$K_{FFT}(k_x, k_y) = \eta(k_x, k_y)\sqrt{S(k_x, k_y)/2} \tag{S13}$$

The division by 2 accounts for allocating power to both positive and negative frequencies for a real-valued field. The zero-frequency component $K_{FFT}(0, 0)$ is set to zero to enforce a zero mean for the generated field.

(v) **Inverse FFT:** Obtain the real-space GRF sample $K(\mathbf{x})$ by applying the inverse Fast Fourier Transform (IFFT) and taking the real part:

$$K(\mathbf{x}) = \mathrm{Re}\left[\mathrm{IFFT}_{2D}\{K_{FFT}(k_x, k_y)\}\right] \tag{S14}$$

This procedure is repeated M times to generate the dataset $\{K^{(m)}\}_{m=1}^M$.

2.2. Data Preprocessing for Autoencoder Training

Before being fed into the autoencoder, the raw GRF samples undergo several preprocessing steps as implemented in the code:

i. **Standardization:** Global mean μ and standard deviation σ are computed across all pixels of all scaled samples. The fields are then standardized: $K^{(m)}_{std} = (K^{(m)}_{scaled} - \mu)/(\sigma + \epsilon)$, where ϵ is a small constant for numerical stability.

ii. **Normalization to [0, 1]:** Global minimum $K_{min,std}$ and maximum $K_{max,std}$ are found across all standardized samples. The fields are then normalized to the range $[0, 1]$:

$$K^{(m)}_{norm} = \frac{K^{(m)}_{std} - K_{min,std}}{K_{max,std} - K_{min,std} + \epsilon} \tag{S15}$$

This final normalization step produces the input data $K^{(m)}_{norm}$ for the autoencoder. A channel dimension is added, resulting in input tensors of shape $[M, N, N, 1]$.

2.3. Autoencoder Architecture

This Supporting Information provides further technical details on the autoencoder architecture employed in Scenario 2 for generating parameterized hydraulic conductivity tensors $K(\mathbf{x};\boldsymbol{\lambda})$ within the physics-informed neural network (PINN) framework. The autoencoder serves to learn a low-dimensional latent representation $\boldsymbol{\lambda}$ from a dataset of high-dimensional Gaussian Random Field (GRF) samples, enabling efficient parameterization of complex geological structures.

The autoencoder consists of an encoder E_{ϕ_1} and an Implicit Neural Representation (INR) decoder (coordinate-based decoder) D_{ϕ_2}.

2.3.1. Encoder Architecture

The encoder network E_{ϕ_1} maps an input conductivity field realization K, typically discretized on an $N \times N$ grid (input shape $[N, N, 1]$), to a low-dimensional latent vector $\boldsymbol{\lambda} = E_{\phi_1}(K)$. The architecture adheres to a standard Convolutional Neural Network (CNN) design typical of autoencoder frameworks. It comprises a series of convolutional layers using 3×3 kernels with a stride of 2. This stride configuration performs spatial downsampling, effectively halving the grid resolution at each step while simultaneously increasing the number of feature channels. Normalization layers and ReLU activation functions are applied after each convolution to stabilize training and introduce non-linearity.

Upon passing through the convolutional blocks, the resulting feature map is flattened (i.e., reshaped into a one-dimensional vector) and processed by a sequence of fully connected dense layers with ReLU activations. The final layer projects this vector down to the target latent dimension d_λ via a linear activation. In this study, we set $d_\lambda = 2$. This choice represents a deliberate trade-off: while higher dimensions would increase the representational power of the autoencoder, we prioritized a compact parameterization to ensure computational tractability for the downstream PINN solver. We selected the minimum dimension sufficient to capture the essential heterogeneity, thereby limiting the dimensionality of the PINN's input space while also facilitating direct visualization. The weights and biases of these layers constitute the parameter set ϕ_1.

2.3.2. INR (coordinate-based) Decoder Architecture

The INR decoder network D_{ϕ_2} adopts a resolution-invariant approach, differing from typical deconvolutional architectures. Instead of reconstructing a fixed-size grid, it functions as an implicit neural representation Sitzmann et al. [2020]. It takes a specific latent vector $\boldsymbol{\lambda} \in \mathbb{R}^{d_\lambda}$ and a set of arbitrary spatial query coordinates $\mathbf{X}_{query} = \{\mathbf{x}_j = (x_j, y_j)\}_{j=1}^{N_q}$ as input, and outputs the corresponding predicted conductivity values $\{\hat{K}(\mathbf{x}_j;\boldsymbol{\lambda})\}_{j=1}^{N_q}$ at those locations. This design is particularly advantageous as it allows the trained decoder to generate conductivity fields at any desired spatial resolution during the subsequent PINN training phase, independent of the resolution used for autoencoder training. The architecture combines positional encoding of coordinates with a Multi-Layer Perceptron (MLP).

Positional Encoding of Coordinates. To effectively incorporate spatial information into the MLP, the continuous input coordinates $\mathbf{x} = (x, y)$ (normalized to $[0, 1]$) are first mapped to a higher-dimensional feature space using a positional encoding (PE) function $\gamma(\cdot)$, similar to techniques used in NeRF Tancik et al. [2020] and Transformers Vaswani et al. [2017]. This encoding helps the MLP capture high-frequency details in the spatial domain. Following the implementation, we define a set of N_{PE} frequency multipliers (bands) $B = \{b_k\}_{k=1}^{N_{PE}}$. For a single coordinate component x, the PE is calculated as:

$$\gamma(x) = [\cos(2\pi b_1 x), \sin(2\pi b_1 x), \dots, \cos(2\pi b_{N_{PE}} x), \sin(2\pi b_{N_{PE}} x)]^T \in \mathbb{R}^{2N_{PE}} \tag{S16}$$

The full positional encoding for the coordinate vector $\mathbf{x} = (x, y)$ is obtained by concatenating the encodings for each component:

$$\gamma(\mathbf{x}) = \begin{bmatrix} \gamma(x) \\ \gamma(y) \end{bmatrix} \in \mathbb{R}^{4N_{PE}} \tag{S17}$$

This results in a fixed-size, high-dimensional representation of the spatial location, irrespective of the number of query points.

MLP for Conductivity Prediction. For each query coordinate $\mathbf{x}_j$ within a batch associated with a latent vector $\boldsymbol{\lambda}$, the INR decoder first computes its positional encoding $\gamma(\mathbf{x}_j)$. This encoded coordinate vector is then concatenated with the latent vector $\boldsymbol{\lambda}$. This combined feature vector serves as the input to a deep MLP:

$$\mathbf{z}_j = \text{concat}(\boldsymbol{\lambda}, \gamma(\mathbf{x}_j)) \in \mathbb{R}^{d_\lambda + 4N_{PE}} \tag{S18}$$

The MLP, comprising the bulk of the decoder parameters ϕ_2, consists of a sequence of dense layers with ReLU activation functions, transforming the input $\mathbf{z}_j$ through progressively learned feature representations $\mathbf{o}_j^{(l)}$:

$$\mathbf{o}_j^{(l+1)} = \text{ReLU}(\mathbf{W}^{(l)}\mathbf{o}_j^{(l)} + \mathbf{b}^{(l)}), \quad \text{with } \mathbf{o}_j^{(0)} = \mathbf{z}_j \tag{S19}$$

The specific layer widths used in our implementation ($[8, 32, 128, 256, 256, 128, 32, 8]$ neurons per layer) define the capacity of the network to model the mapping from the latent space and coordinates to the conductivity value.

Denormalization. A final dense layer with a single output neuron and a suitable activation function σ_{out} ('hard_sigmoid' as implemented, which clips the output to $[0, 1]$) produces the normalized predicted conductivity value:

$$\hat{K}_{norm}(\mathbf{x}_j;\boldsymbol{\lambda}) = \sigma_{out}(\mathbf{W}_{out}\mathbf{o}_j^{(L)} + b_{out}) \in [0, 1] \tag{S20}$$

where L is the number of hidden layers in the MLP.

Finally, the normalized output $\hat{K}_{norm}$ is mapped back to the physical domain. We note that while the preprocessing (Section 2.2) consists of sequential standardization and normalization steps, both are linear operations. Consequently, their inverse is applied here as a single composite affine transformation that maps the network output directly from $[0, 1]$ to the physical range of the hydraulic conductivity $[K_{min}, K_{max}]$:

$$\hat{K}(\mathbf{x}_j; \boldsymbol{\lambda}) = (K_{max} - K_{min}) \cdot \hat{K}_{norm}(\mathbf{x}_j; \boldsymbol{\lambda}) + K_{min} \tag{S21}$$

In our specific experimental setup, where the bounds are defined by $K_{min} = e^{-0.8}$ and $K_{max} = e^{0.8}$, this yields:

$$\hat{K}(\mathbf{x}_j; \boldsymbol{\lambda}) = (e^{0.8} - e^{-0.8}) \cdot \hat{K}_{norm}(\mathbf{x}_j; \boldsymbol{\lambda}) + e^{-0.8}. \tag{S22}$$

This final value $\hat{K}(\mathbf{x}_j; \boldsymbol{\lambda})$ is the hydraulic conductivity used by the PINN solver when evaluating the physics-informed loss for parameter vector $\boldsymbol{\lambda}$ at location $\mathbf{x}_j$.

2.4. Autoencoder Training Objective

The autoencoder parameters $\phi = (\phi_1, \phi_2)$ are optimized jointly by minimizing a composite loss function $\mathcal{L}_{AE}$. This function is designed to ensure accurate reconstruction while preserving key statistical and structural properties of the input fields. The total loss is defined as a weighted sum of five components:

$$\mathcal{L}_{AE} = w_{recon}\mathcal{L}_{recon} + w_{mean}\mathcal{L}_{mean} + w_{var}\mathcal{L}_{var} + w_{freq}\mathcal{L}_{freq} + w_{smooth}\mathcal{L}_{smooth} \tag{S23}$$

where the weights are set to $(w_{recon}, w_{mean}, w_{var}, w_{freq}, w_{smooth}) = (50, 50, 50, 10, 50)$ in our implementation. The individual components are defined as follows:

Weighted Reconstruction Loss ($\mathcal{L}_{recon}$). We employ a modified Mean Squared Error (MSE) that emphasizes accuracy near the extrema (minimum and maximum values) of the conductivity field. We first define a spatial weight map $w^{(m)}(\mathbf{x})$ inversely proportional to the distance from the global bounds:

$$w^{(m)}(\mathbf{x}) \propto \frac{1}{\epsilon + \min\left(|K^{(m)}_{norm}(\mathbf{x}) - K^{(m)}_{min}|, |K^{(m)}_{norm}(\mathbf{x}) - K^{(m)}_{max}|\right)}, \tag{S24}$$

which is normalized such that its spatial mean is 1. The loss is then the weighted MSE:

$$\mathcal{L}_{recon} = \mathbb{E}\left[w^{(m)}(\mathbf{x})\left(\hat{K}^{(m)}_{norm}(\mathbf{x}) - K^{(m)}_{norm}(\mathbf{x})\right)^2\right]. \tag{S25}$$

Statistical Moment Losses ($\mathcal{L}_{mean}$ and $\mathcal{L}_{var}$). To ensure the generative model captures the global statistics of the field, we penalize deviations in the spatial mean (μ) and variance (σ^2):

$$\mathcal{L}_{mean} = \mathbb{E}\left[(\mu_{K^{(m)}} - \mu_{\hat{K}^{(m)}})^2\right], \tag{S26}$$

$$\mathcal{L}_{var} = \mathbb{E}\left[(\sigma^2_{K^{(m)}} - \sigma^2_{\hat{K}^{(m)}})^2\right]. \tag{S27}$$

Frequency Domain Loss ($\mathcal{L}_{freq}$). This term ensures the spectral properties of the field are preserved by minimizing the difference in the magnitude of the 2D Fast Fourier Transform (FFT) coefficients:

$$\mathcal{L}_{freq} = \mathbb{E}\left[|\text{FFT}(\hat{K}^{(m)}_{norm}) - \text{FFT}(K^{(m)}_{norm})|\right]. \tag{S28}$$

Smoothness Loss ($\mathcal{L}_{smooth}$). To encourage physical consistency and spatial smoothness, we penalize the magnitude of the first- and second-order spatial gradients (Laplacian smoothness) of the reconstructed field:

$$\mathcal{L}_{smooth} = \|\nabla \hat{K}^{(m)}_{norm}\|^2 + \|\nabla^2 \hat{K}^{(m)}_{norm}\|^2. \tag{S29}$$

The optimization is performed using the Adam optimizer Kingma and Ba [2017] with an exponential decay learning rate schedule.

2.4.1. PINN Solver with Explicit Conditioning Parameterization Scheme

Once the Autoencoder is trained, its parameters are stored, particularly those related to the INR decoder D_{ϕ_2}. These parameters are needed to train the PirateNet-based PINN solver $u_{\boldsymbol{\Theta}_\lambda}$. In Scenario 2, the parameter vector for the PINN is the latent vector $\boldsymbol{\lambda} \in \Lambda \subset \mathbb{R}^{d_\lambda}$. We typically sample $\boldsymbol{\lambda}$ from the distribution induced by the encoder on the training data, $p(\boldsymbol{\lambda}) \approx p(E_{\phi_1}(K))$, or a fitted approximation (e.g., a multivariate Gaussian distribution fitted to the encoded vectors $\{\boldsymbol{\lambda}^{(m)}\}_{m=1}^{M}$).

The input to the PirateNet solver $u_{\boldsymbol{\Theta}_\lambda}$ is the concatenated vector $(x, y, \boldsymbol{\lambda})$. Crucially, the hydraulic conductivity field $K(\mathbf{x}; \boldsymbol{\lambda})$ required within the PINN's loss function (Eq. 17) is now defined by the pre-trained decoder:

$$K(\mathbf{x}; \boldsymbol{\lambda}) = \tilde{D}_{\phi_2}(\boldsymbol{\lambda}, \mathbf{x}) \tag{S30}$$

where $\tilde{D}_{\phi_2}$ represents the decoder function, including the final denormalization step to scale the output to the physical conductivity range (e.g., $[e^{-.8}, e^{.8}]$).

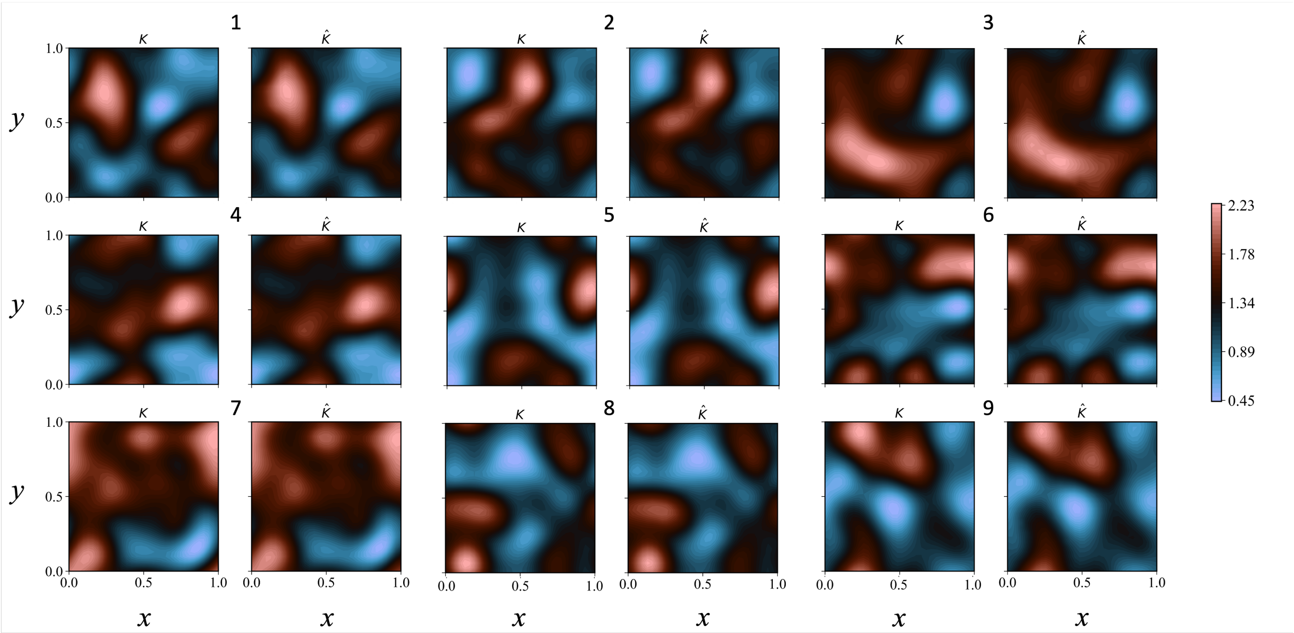

Fig. S2. Qualitative assessment of the autoencoder reconstruction capability. Nine representative pairs of original conductivity fields (K) from the training dataset and their corresponding reconstructions ($\hat{K}$) by the trained INR decoder. Each numbered pair displays the ground truth field alongside the field reconstructed by the decoder from its 2D latent representation. The color bar indicates the magnitude of the normalized hydraulic conductivity tensor values. The high degree of visual similarity confirms the decoder ability to generate complex heterogeneous fields.

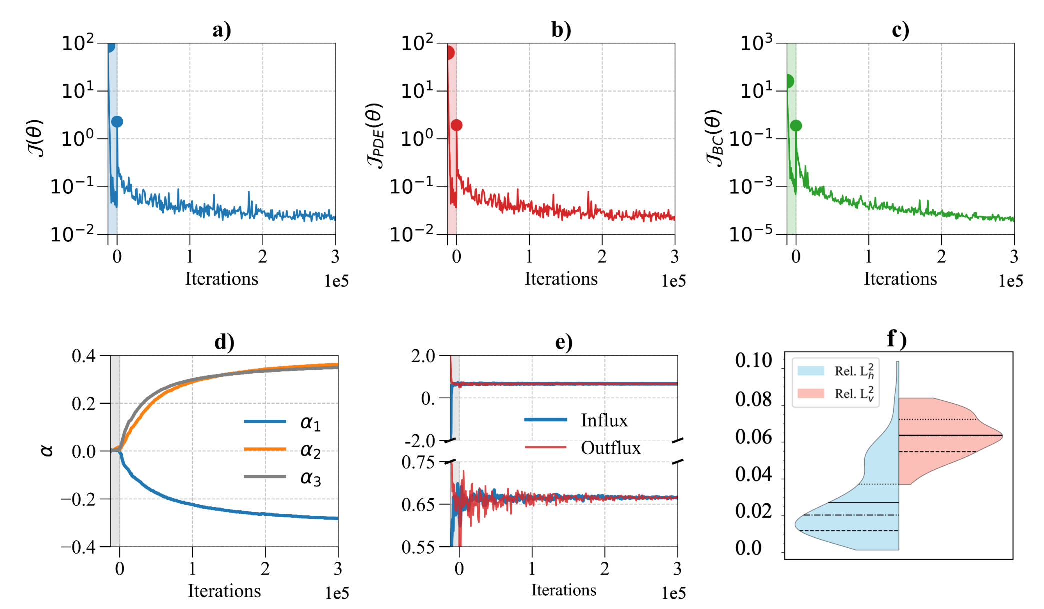

Fig. S3. Training dynamics of Scenario 2. Evolution of (a) the total physics-informed loss $\mathcal{J}(\theta)$, (b) the PDE residual component $\mathcal{J}_{PDE}(\theta)$, and (c) the boundary condition residual component $\mathcal{J}_{BC}(\theta)$. (d) Evolution of the trainable α_i $(i = 1, 2)$ parameters associated with the adaptive skip connections. (e) Convergence of the mean influx and outflux, the two reported plots display results with different vertical axis scale. The narrow shaded region in panels (a)-(e) corresponds to the initial warm-up phase where the network is trained on the mean of the latent parameter distribution before training on the full sampled latent space. Filled circles in panels (a)-(c) indicate the starting point for each iteration of the training phase (f) Violin plots summarizing the distribution of relative L^2 errors for computed hydraulic heads (Rel. $L^2_{\hat{h}}$, light blue) and auto-differentiated velocity (Rel. $L^2_{\hat{\mathbf{v}}}$, salmon) over 512 test realizations sampled from the latent space, compared to FEM reference solutions.

.

Figure S2 displays nine pairs of reference conductivity fields alongside their corresponding reconstructions obtained through the INR decoder from their 2D latent representations. The approximated fields display a striking similarity to the reference ones also in terms of fine-scale details.

During the PINN training (see Section 2), for a sampled point $(\mathbf{x}_i, \boldsymbol{\lambda}_i)$:

i $K(\mathbf{x}_i; \boldsymbol{\lambda}_i)$ is computed by passing $\boldsymbol{\lambda}_i$ and $\mathbf{x}_i$ through the decoder D_{ϕ_2} (Eq. S30).
ii The spatial derivatives $\nabla_{\mathbf{x}} K(\mathbf{x}_i; \boldsymbol{\lambda}_i)$ needed for the PDE residual are obtained by applying AD through the computational graph of the decoder D_{ϕ_2} with respect to the spatial inputs $\mathbf{x}_i$.

This approach allows the PINN solver $u_{\boldsymbol{\Theta}_\lambda}$ to learn the mapping from the low-dimensional latent representation $\boldsymbol{\lambda}$ and spatial coordinates $\mathbf{x}$ to the corresponding head and velocity fields. The use of the INR decoder D_{ϕ_2} renders the definition of K continuous and independent of the discretization mesh. This implies the PINN can be trained on arbitrary collocation points not bound to the original training grid, while the spectral content of the reconstructed field remains effectively bounded by the resolution of the data used to train the autoencoder. The performance of the training method is illustrated in Figure S3.

I

3. FEM as baseline

Solutions obtained with the Finite Element Method (FEM) are considered as a baseline reference to validate our PINN results. Our FE approach is based on a standard implementation, here recalled for completeness. The FEM is based on the weak (or variational) formulation of the governing PDE system. By combining the continuity equation (Eq. 7a) and Darcy's law (Eq. 7b), we obtain:

$$-\nabla \cdot (K(\mathbf{x}; \boldsymbol{\lambda}) \nabla h(\mathbf{x}; \boldsymbol{\lambda})) = f(\mathbf{x}), \quad \forall \mathbf{x} \in \Omega. \tag{S31}$$

The weak form is derived by multiplying this equation by a suitable test function ψ and integrating over the domain Ω. Applying Green's first identity (integration by parts) yields the integral form:

$$\int_\Omega K(\mathbf{x}; \boldsymbol{\lambda}) \nabla h \cdot \nabla \psi \, d\mathbf{x} = \int_\Omega f \psi \, d\mathbf{x} - \int_{\partial\Omega} \psi (\mathbf{v} \cdot \mathbf{n}) \, d\mathbf{s}. \tag{S32}$$

Following this formulation, one finds a solution $h \in \mathcal{V}$ such that Eq. S32 holds for all test functions $\psi \in \mathcal{V}_0$. For this problem, the trial (or solution) space $\mathcal{V}$ and the test space $\mathcal{V}_0$ are defined as specific Sobolev spaces. The solution space is $\mathcal{V} = H^1(\Omega)$, the space of functions with square-integrable first derivatives. The test space is a subspace of $\mathcal{V}$ where the functions are zero on the Dirichlet boundary, i.e., $\mathcal{V}_0 = \{\psi \in H^1(\Omega) \mid \psi|_{\Gamma_D} = 0\}$.

For our numerical simulations, we employ the FEniCS Project finite element software library [Alnæs et al., 2015] to implement a standard continuous Galerkin method. The computational domain, $\Omega = [0, 1]^2$, is discretized using a uniform quadrilateral grid, which is then split into triangular elements. To ensure a highly-resolved reference solution, while considering computational limits and efficiency, we rely on a 200×200 grid (160, 801 elements) for both Case Studies. Hydraulic head within each element is approximated using second-order Lagrange basis functions (P_2 elements, corresponding to the 'CG=2' setting in FEniCS). This choice of a high-order basis function provides a more accurate representation of the solution for a given mesh density. Suitability of this mesh resolutions is confirmed by a mesh convergence analysis (details are offered in Supporting Information 3.1).

The discretization results in a large, sparse system of linear equations which is solved to obtain the nodal values of the hydraulic head. The corresponding velocity field is subsequently recovered by computing the gradient of the finite element solution. These simulations serve as a reliable ground truth for assessing the accuracy of our PINN-based surrogate model.

3.1. FEM Mesh Convergence Analysis

To ensure the reliability of the Finite Element Method (FEM) simulations used as a ground truth, we performed a mesh convergence study. The objective was to select a mesh discretization that provides a high-fidelity solution while remaining computationally tractable for the full ensemble study. This analysis is critical for establishing that our chosen mesh resolution is well within the convergent regime, thereby minimizing the influence of numerical discretization error.

The analysis was conducted for representative realizations of both the Gaussian anomaly (Case 1) and the heterogeneous autoencoder field (Case 2). For each case, the problem was solved on a sequence of progressively refined meshes (25×25, 125×125, 200×200, and 250×250). The solution obtained on the finest achievable mesh (**300 × 300**) within our computational memory limits was used as the reference "ground truth" for this study. The error for each coarser mesh was then computed as the relative L_2 norm of the difference between its solution and this reference.

Figure S4 plots this relative error against the number of degrees of freedom (DOF) for both the hydraulic head (h) and the derived velocity field ($\mathbf{v}$). The results demonstrate the expected asymptotic convergence behavior. The error in the hydraulic head (the primary variable) converges rapidly and monotonically for both scenarios. The error in the velocity field, a derived quantity, converges more slowly which is a known challenge in numerical simulations, particularly for the complex heterogeneous field (Panel b).

Crucially, the plots reveal a point of diminishing returns. Beyond approximately 1.6×10^5 Degrees of Freedom (corresponding to a mesh of roughly 200×200). Further refinement offers negligible improvement on the acceptable 1% discretization error in velocity, while dramatically increasing computational cost. Therefore, we selected a **200 × 200** mesh as the basis for generating our reference solutions. This represents a pragmatic trade-off, providing a ground truth that is demonstrably in the convergent regime and highly accurate, without being compromised by the numerical instabilities or prohibitive costs of extreme mesh refinement.

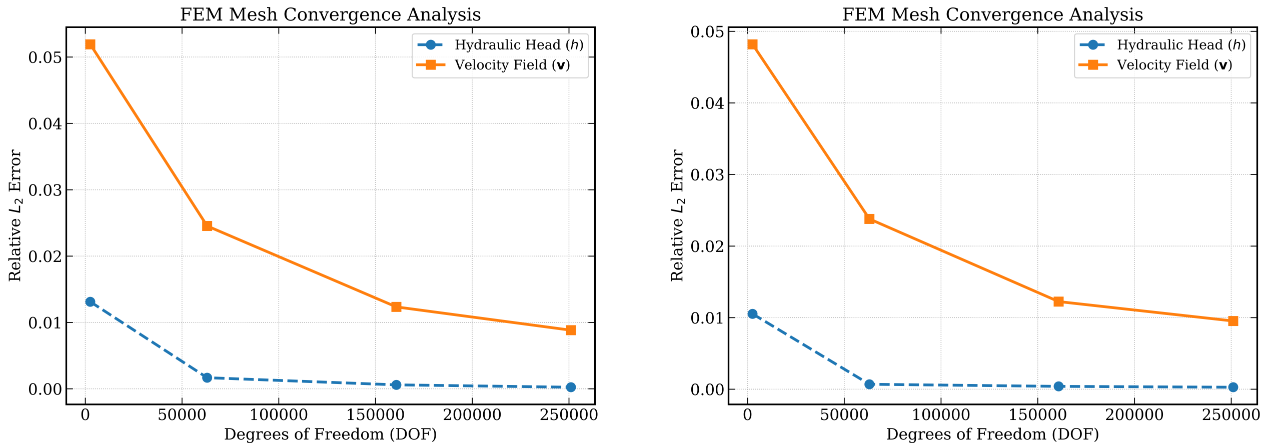

Fig. S4. Mesh convergence results for the FEM solver. The plots show the relative L_2 error of the hydraulic head and velocity fields as a function of mesh refinement (DOF), relative to a high-fidelity reference solution. **(a)** Convergence for scenario 1. **(b)** Convergence for a representative heterogeneous field from scenario 2. The results justify the selection of a mesh with $\approx 10^5$ DOF as an optimal trade-off between accuracy and stability.

4. Comparison with Physics-Informed Neural Operators (PINO)

We compare our PINN-based approach against a state-of-the-art Physics-Informed Neural Operator (PINO). To ensure a fair, ”zero-data” comparison equivalent to our methodology, the PINO was trained exclusively on grid-based physics residuals without any pre-computed FEM target data. We remark that this comparison is not to demonstrate which approach is superior in general terms, but to provide further motivation for the modeling choices that distinguish our approach with respect to PINOs.

4.1. PINO Architecture and Zero-Data Training Formulation

We implemented the standard PINO architecture, as introduced by Li et al. [2024], which builds on the Fourier Neural Operator (FNO) backbone [Li et al., 2021].

For this benchmark on Scenario 1 (Gaussian anomaly), the architecture utilized 4 Fourier blocks, 16 Fourier modes per spatial dimension, a latent channel width of 64, and GeLU activations. As recommended in the foundational FNO literature for non-periodic domains [Li et al., 2021], zero-padding was applied to the spatial domain prior to the spectral convolutions to mitigate periodic boundary artifacts. The network was trained on a fixed grid resolution of 128×128.

Crucially, because PINO outputs the solution on discrete grids, the exact Automatic Differentiation (AD) used in our PINN cannot be applied directly. Instead, following previous literature, the velocity field ($\hat{\mathbf{v}} = -K\nabla\hat{h}$) and the governing PDE residual ($\nabla \cdot \hat{\mathbf{v}} = 0$) were computed using staggered-grid central finite differences. The model was optimized using the Adam optimizer for 200,000 iterations.

4.2. Evaluation of Physical Fidelity and Resolution Invariance

We tested resolution invariance, i.e. the ability to train on a coarse grid and perform zero-shot inference on finer grids. We evaluated the 128×128-trained PINO across three ensembles of 512 test realizations, each at a distinct resolution: 64×64, 128×128, and 256×256. To isolate the operator's native predictive capabilities from secondary numerical artifacts, the PINO predictions were compared against FEM solutions explicitly generated at each matching target resolution. The results are detailed in Figure S5.

Native Resolution Performance At its native training resolution (128×128, Figure S5b), the PINO captures the global topological features of the hydraulic head with a maximum relative L^2 error of approximately 3.9%. However, the relative error for the derived velocity field is markedly higher, reaching up to 15.1%. In the study by Li et al.[Li et al., 2024] evaluations related to the Darcy problem focused on the scalar head variable; however, our analysis reveals that extracting derived gradient quantities (velocity) on a discrete grid can lead to a deterioration of accuracy.

Test at multiple resolutions When the trained PINO is evaluated at resolutions different from its training grid, its predictive accuracy decreases. As observed in Figure S5a (64×64) and Figure S5c (256×256), the maximum relative error for the hydraulic head and the velocity error exceed 24% and 100%, respectively.

This degradation of the accuracy highlights a critical vulnerability of applying grid-based operator learning without supervised data. Our results suggest that PINO likely overfits to the specific numerical truncation errors of the finite-difference stencils at the 128×128 resolution. When the inference grid is altered, the numerical dispersion of the finite difference operators changes, likely causing the learned spectral weights to unbalance the PDE residuals.

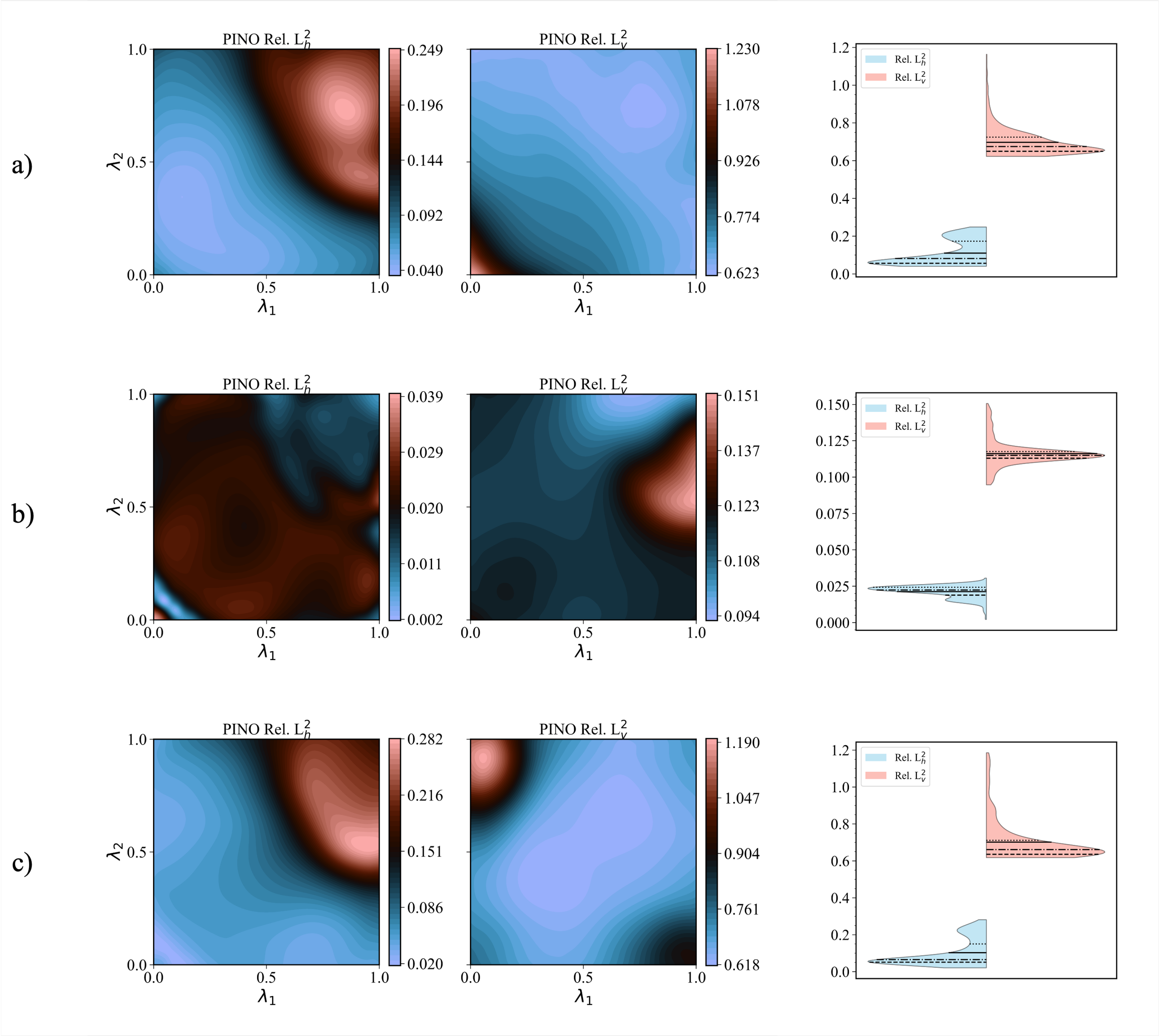

Fig. S5. Resolution Invariance and Fidelity Analysis of the Physics-Informed Neural Operator (PINO). The PINO was trained exclusively on a 128 × 128 grid and subsequently evaluated across three resolutions: **(a)** 64 × 64, **(b)** 128 × 128, and **(c)** 256 × 256. Contour maps display the spatial distribution of the relative L^2 error for hydraulic head (left) and velocity (center). Violin plots (right) summarize the error distributions across an ensemble of 512 test realizations.

4.3. Discussion

Our benchmark highlights a fundamental difference between our approach and PINOs. Our proposed approach — leveraging a coordinate-based PINN and exact AD — is strictly continuous and mesh-free. Our results suggest that grid-based PINOs could face limitations when training without labeled data, particularly in the presence of heterogeneous parameter fields. We are also aware that the field of operator learning is rapidly evolving with specialized, problem-specific modifications that can likely resolve or mitigate the observed behaviors.

An analysis by Noguer i Alonso [2025] discussed the trade-off between the *solution-specific accuracy* of PINNs and the *operator-level generalization* of Physics-Informed Neural Operators (PINOs) [Li et al., 2024]. PINNs excel at yielding high-fidelity solutions for specific problem instances by embedding the governing PDE directly into the loss function. In contrast, PINOs are designed for efficiency in parametric studies. They achieve this by training a neural operator on a combination of available input-output simulation data and a physics-based loss term computed on collocation points. The physics-based loss acts as a regularizer, enabling the PINO to learn from a sparser set of simulation data than a purely data-driven operator would require. However, this approach still prioritizes operator-level generalization and may sacrifice the pinpoint accuracy that a solution-specific PINN can achieve for any single instance.

In our work, we explore how to advance the PINN framework itself to address two of its primary challenges. The first is the issue of parameter scalability: a standard PINN is architecturally a solver for a single problem instance and requires a complete, independent re-training for each new parameter value, making it inefficient for parametric studies. The second is the presence of known training pathologies, such as spectral bias and vanishing gradients, which can hinder convergence. By addressing these challenges, we aim to develop a framework that retains the high physical fidelity of a direct solver while achieving the parametric flexibility of a learned operator. This positions our work as a principled, physics-grounded alternative to purely data-driven regularization.

5. Computational Efficiency, Amortized Cost, and Scaling Behavior

A fundamental motivation for developing differentiable surrogate solvers is the acceleration of many-query applications, such as Monte Carlo-based Uncertainty Quantification (UQ) and inverse modeling. To systematically assess the computational efficiency of the proposed parameterized PINN, we evaluate its performance through the paradigm of amortized computational cost, dividing the computational burden into an upfront *offline phase* (model training) and an *online phase* (inference per parameter realization).

Performance metrics were recorded using a single NVIDIA GeForce RTX 4090 GPU for the PINN and a 13th Gen Intel(R) Core(TM) i9-13900KF CPU for the Finite Element Method (FEM) baseline. For the FEM baseline, the computational domain was discretized into a 200×200 highly-resolved mesh, requiring approximately 14.0 seconds to assemble and solve the linear system for a single realization. By contrast, the PINN inference merely requires a forward pass of the coordinate queries through the neural network.

Table S1 summarizes the computational costs for both Scenario 1 (Functional Parameterization) and Scenario 2 (Autoencoder-based Parameterization). The PINN demands a significant offline investment: Scenario 1 trained for 3×10^5 epochs in approximately 10.4 hours, while the more complex Scenario 2 required approximately 14.5 hours. However, the online inference time for the PINN is orders of magnitude faster, taking approximately 0.1 seconds to evaluate the full high-resolution spatial grid for a new parameter instance $\boldsymbol{\lambda}$.

Table S1. Amortized Computational Cost Comparison. Offline training times and online inference times (per realization) for the FEM baseline and the proposed PINN frameworks. The projected total time for a standard Monte Carlo UQ task (10^4 realizations) highlights the break-even efficiency of the surrogate.

**Method**	**Offline Phase (Training Time)**	**Online Phase (Inference per Sample)**	**Total Time for 10^4 MC Samples**
FEM Baseline (200×200)	0.0 hours	≈ 14.00 s	≈ 38.8 hours
PINN - Scenario 1 ($d_\lambda = 2$)	≈ 10.4 hours	≈ 0.10 s	≈ 10.7 hours
PINN - Scenario 2 ($d_\lambda = 2$)	≈ 14.5 hours	≈ 0.15 s	≈ 14.9 hours

This dichotomy creates a clear computational *break-even point*. For Scenario 1, the cumulative time of training and querying the PINN becomes lesser than the equivalent FEM evaluations after approximately $2,700$ realizations. For a typical forward Monte Carlo UQ task requiring 10^4 samples, the FEM solver would demand nearly 39 hours of continuous computation. In contrast, the trained PINN generates the entire 10^4 ensemble in mere minutes, reducing the total end-to-end time to approximately 10.7 hours—a nearly 4-fold acceleration that grows linearly with the number of required samples.

Scaling Behavior with Parameter Dimensionality. A critical aspect of parameterized surrogate modeling is its scaling behavior with respect to the dimensionality of the latent parameter space (d_λ). In traditional numerical solvers, handling higher-dimensional parametric uncertainty heavily exacerbates the computational cost, as the number of forward evaluations required to adequately sample the manifold grows exponentially.

For the proposed PINN solver, the online inference time scales highly efficiently, as increasing d_λ merely adds a marginal number of input neurons to the lifting layer, resulting in an $O(1)$ impact on evaluation speed. During the offline training phase, our framework exploits a continuous resampling strategy: by randomly drawing new Sobol samples of $\boldsymbol{\lambda}$ at every training epoch, the model effectively explores the continuous parameter space without requiring an exponential increase in the batch size or GPU memory footprint.

However, we emphasize that this formulation does not entirely circumvent the curse of dimensionality. As d_λ increases to represent more complex geological structures (e.g., shorter correlation lengths), the volume of the solution manifold expands exponentially. Consequently, learning this complex mapping fundamentally challenges the optimization dynamics. Scaling to higher-dimensional latent spaces will inevitably necessitate wider and deeper network architectures, proportionally longer offline training regimes, and potentially more sophisticated loss-balancing heuristics to maintain acceptable physical fidelity. Thus, rather than a definitive replacement for traditional methods in high-dimensional UQ, we position this differentiable solver framework as a complementary step within the Scientific Machine Learning (SciML) community, offering distinct advantages specifically in many-query regimes where the upfront training cost can be effectively amortized.

6. Ablation Study: Architecture and Optimization Strategies

Our proposed parameterized differentiable solver integrates several advanced Scientific Machine Learning (SciML) components, including Random Fourier Features (RFF), PirateNet adaptive residual connections, gradient balancing, and a multi-stage curriculum learning strategy.

The necessity of Fourier embeddings to overcome spectral bias [Tancik et al., 2020] and adaptive residual connections to mitigate vanishing gradients in deep Physics-Informed Neural Networks [Wang et al., 2024] have been exhaustively ablated and proven in their respective foundational works. Consequently, our architecture adopts this state-of-the-art base engine to focus specifically on the novel challenges of learning continuous parametric solution manifolds in zero-data regimes. To quantify the impact of our specific optimization contributions, we conducted targeted ablation studies on Scenario 1 (Gaussian Anomaly).

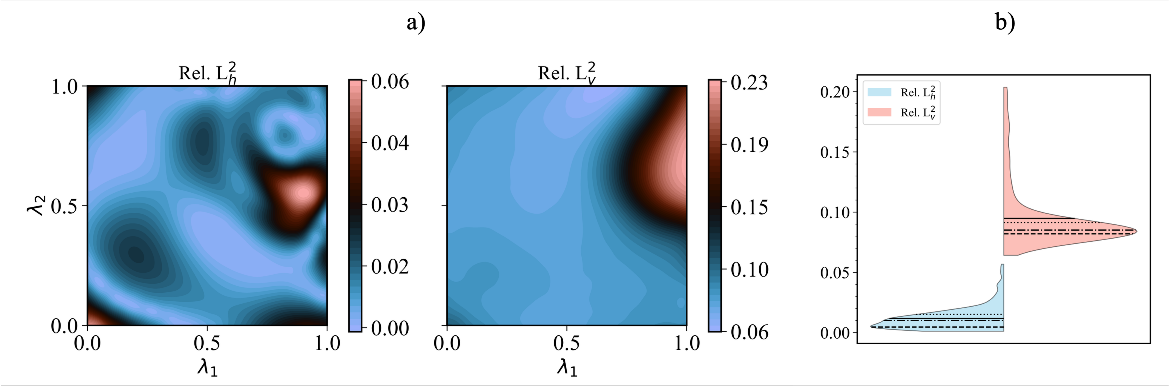

Fig. S6. Ablation of the Multi-Stage Curriculum Learning Strategy (Scenario 1). Performance of the parameterized PINN trained directly on the full parameter space without the stage-wise pre-training curriculum. **(a)** Contour maps of the relative L^2 error for the predicted hydraulic head (left) and velocity field (right) across the $\boldsymbol{\lambda} = (\lambda_1, \lambda_2)$ parameter space. While the spatial distribution of the error remains qualitatively similar to the baseline model, the lack of curriculum training causes the optimizer to settle into a poorer local minimum, amplifying the maximum velocity error to over 23% in the most challenging regions ($\lambda_1 > 0.6$). **(b)** Violin plots summarizing the error distributions across 512 test realizations. Compared to the robust baseline model (Figures 3f and 5 in the main text), the ablated model exhibits a broader, heavier-tailed error distribution.

6.1. Ablating the Multi-Stage Curriculum Learning

The core idea underpinning our multi-stage curriculum is to allow the network to learn the fundamental solution behavior in a simplified parameter setting (the mean parameter field) before exposing it to the full variability of the high-dimensional parameter space.

To evaluate the necessity of this strategy, we trained an identical network architecture with all other features (RFF, PirateNet, Gradient Balancing) enabled, but strictly removed the curriculum. The network was forced to train on the full $[0, 1] \times [0, 1]$ spatial and parametric domains simultaneously from Epoch 1.

As illustrated in Figure S6, omitting the multi-stage curriculum noticeably degrades the generalization performance across the parameter space. The spatial error contours (Figure S6a) indicate that while the ablated model struggles in the same parametric regions as the baseline model (specifically when the Gaussian anomaly approaches the outflow boundary, $\lambda_1 > 0.6$), the magnitude of the error is significantly amplified. The maximum relative L^2 error for the derived velocity field increases from approximately 8% (in our proposed model; see Figure 5 in the main text) to over 23%. This degradation is further reflected in the violin plots (Figure S6b), which display a broader, heavier-tailed error distribution. These results demonstrate that directly optimizing over the full, complex parameter space from a random initialization leaves the network susceptible to suboptimal local minima.

The efficacy of our proposed curriculum can be heuristically understood through the lens of recent work on phase transitions in PINN training [Anagnostopoulos et al., 2026]. This work identifies a critical phase transition into a "diffusion equilibrium" (DE), marked by an abrupt drop in test error and stable optimizer convergence. This transition occurs when sample-wise gradients become highly aligned and PDE residuals become homogeneous across the sample space.

Training a parameterized PINN from a random initialization on a highly variable manifold forces the optimizer into a prolonged and chaotic "diffusion" phase, struggling with disordered gradients. Without the curriculum, the optimizer becomes permanently trapped in poor local minima for certain parameter regions. By contrast, our Stage 1 pre-training guides the network to a state that establishes a baseline of residual homogeneity. Consequently, when fine-tuning begins on the full parameter space, the network initializes from a well-conditioned state already close to the DE phase. By progressively expanding the parameter space, our method ensures that the optimizer efficiently navigates the complex loss landscape, maintaining ordered gradients and yielding a robust, globally accurate solution manifold.

6.2. Ablating Gradient Balancing (Bounded GradNorm)

Training Physics-Informed Neural Networks often requires navigating complex optimization landscapes where competing loss terms—such as the domain PDE residual and localized boundary conditions—can exhibit vastly different gradient magnitudes. To

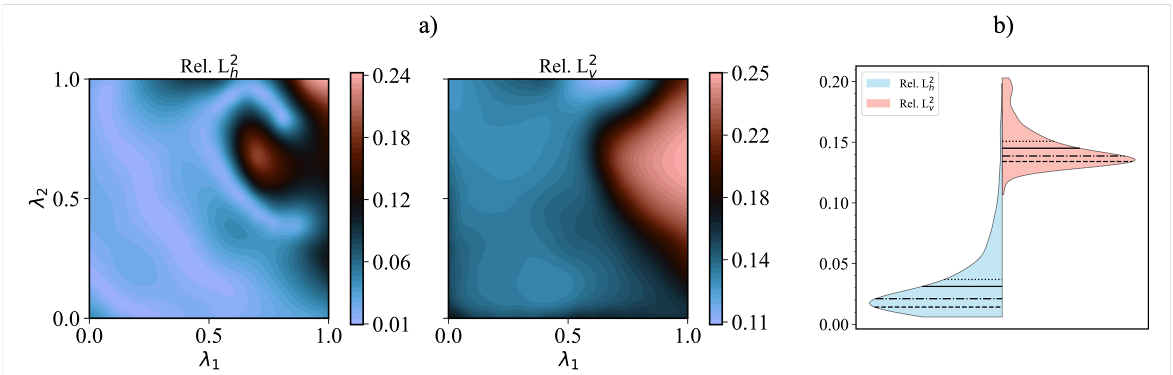

Fig. S7. Ablation of the Gradient Balancing Strategy (Scenario 1). Performance of the parameterized PINN trained with an unweighted sum of the PDE and boundary condition losses. **(a)** Contour maps of the relative L^2 error for the predicted hydraulic head (left) and velocity field (right). The spatial distribution of the error remains qualitatively similar to the baseline model, but the maximum error magnitudes increase to approximately 24% and 25%, respectively. **(b)** Violin plots summarizing the error distributions across 512 test realizations. The upward shift in the velocity error distribution (median ≈ 14%) indicates that the unweighted optimization process yields a less accurate approximation of the solution manifold compared to the adaptively balanced baseline.

address this, our framework employs a Bounded GradNorm adaptive weighting scheme to dynamically balance the backpropagated gradients during training.

To quantify the contribution of this gradient balancing technique, we conducted a second ablation test. The network was trained using the complete multi-stage curriculum and PirateNet architecture, but the gradient balancing is disabled. Consequently, the total loss was formulated as a simple, unweighted sum of the constituent PDE and boundary condition residuals. To ensure that the observed convergence behaviors were not artifacts of a specific random initialization, all ablation configurations in this study were independently trained three times using different random seeds. The resulting error metrics and spatial distributions remained consistent across all runs.

The performance of the unweighted model is presented in Figure S7. The spatial error contours (Figure S7a) indicate that the regions of highest error remain structurally similar to those observed in the baseline model. However, the absence of dynamic gradient weighting leads to a marked increase in the magnitude of these errors. Specifically, the maximum relative L^2 error increases to approximately 24% for the hydraulic head and 25% for the velocity field, compared to the ≈ 8% maximum velocity error achieved by the fully equipped model.

This decrease in overall accuracy is further detailed in the violin plots (Figure S7b). The distribution of the relative velocity error exhibits an upward shift, with the median error resting near 14%. This shift suggests that without an adaptive weighting mechanism, the optimizer struggles to simultaneously satisfy all physical constraints to the same degree of precision across the parametric manifold. These results empirically justify the inclusion of the Bounded GradNorm scheme to maintain a balanced optimization trajectory and achieve higher overall fidelity.

7. Hyperparameter Configurations

This section provides the hyperparameter configurations used for the two case studies presented in the main text [1].

[1] Training is implemented on a single Nvidia GeForce RTX 4090 GPU.

[2] Bounded GradNorm: An adaptive weighting scheme based on GradNorm, which balances the learning rates of different loss components (PDE, Darcy, BCs) by adjusting their weights based on gradient magnitudes. The 'Bounded' variant additionally clips the computed weights or the gradient norms used in the calculation to a predefined range (e.g., [0.1, 10.0]) to prevent excessively large or small weight values and improve stability.

Table S2. Hyperparameter configuration for the parameterized PINN solver in Scenario 1 (Gaussian Bump).

Parameter	Value
**Architecture (PirateNet-inspired)**	
Number of residual blocks (L)	6
Neurons per hidden layer	256
Activation function	Tanh
**Input Embedding**	
Input embedding type	Random Fourier Features
Fourier feature scale	1.0
Random weight factorization	$\mu = .5, \sigma = 0.1$
**Optimizer**	
Optimizer type	Adam Kingma and Ba [2017]
Adam β_1	0.9
Adam β_2	0.999
Adam ϵ	10^{-8}
**Learning Rate Schedule**	
Initial learning rate	10^{-3}
Decay schedule	Exponential Decay
Decay rate	0.9
Decay steps	10,000
Warmup steps	5000
**Training**	
Training steps (Stage 1)	5×10^4
Training steps (Stage 2)	3×10^5
Batch size	4,096
Collocation points per batch	$N_{pde} = 3{,}000$
Boundary points per batch	$N_{bc} = 1{,}096$
**Loss Weighting**	
Weighting scheme	Bounded GradNorm[2]
GradNorm bounds [min, max]	[0.04, 25.0]
Initial loss weights (w_{pde}, w_{bc})	(1.0, 1.0)

References

M. Alnæs, J. Blechta, J. Hake, A. Johansson, B. Kehlet, A. Logg, C. Richardson, J. Ring, M. E. Rognes, and G. N. Wells. *The FEniCS project version 1.5*, page 189–218. De Gruyter, Dec. 2015. doi: 10.1515/9783110359961.189. URL http://dx.doi.org/10.1515/9783110359961.189.

S. J. Anagnostopoulos, J. D. Toscano, N. Stergiopulos, and G. E. Karniadakis. Residual-based attention and connection to information bottleneck theory in pinns, 2023. URL https://arxiv.org/abs/2307.00379.

S. J. Anagnostopoulos, J. D. Toscano, N. Stergiopulos, and G. E. Karniadakis. Learning in pinns: Phase transition, diffusion equilibrium, and generalization. *Neural Networks*, 193:107983, 2026. ISSN 0893-6080. doi: https://doi.org/10.1016/j.neunet.2025.107983. URL https://www.sciencedirect.com/science/article/pii/S0893608025008640.

X. Glorot and Y. Bengio. Understanding the difficulty of training deep feedforward neural networks. In *Proceedings of the thirteenth international conference on artificial intelligence and statistics*, pages 249–256. JMLR Workshop and Conference Proceedings, 2010. URL https://proceedings.mlr.press/v9/glorot10a/glorot10a.pdf.

D. P. Kingma and J. Ba. Adam: A method for stochastic optimization, 2017. URL https://arxiv.org/abs/1412.6980.

Z. Li, N. Kovachki, K. Azizzadenesheli, B. Liu, K. Bhattacharya, A. Stuart, and A. Anandkumar. Fourier neural operator for parametric partial differential equations, 2021. URL https://arxiv.org/abs/2010.08895.

Z. Li, H. Zheng, N. Kovachki, D. Jin, H. Chen, B. Liu, K. Azizzadenesheli, and A. Anandkumar. Physics-informed neural operator for learning partial differential equations. *ACM / IMS J. Data Sci.*, 1(3), May 2024. doi: 10.1145/3648506. URL https://doi.org/10.1145/3648506.

M. Noguer i Alonso. The mathematics of physics-informed neural networks vs. physics-informed neural operators. *SSRN*, 2025. URL https://papers.ssrn.com/sol3/papers.cfm?abstract_id=5297552.

V. Sitzmann, J. N. P. Martel, A. W. Bergman, D. B. Lindell, and G. Wetzstein. Implicit neural representations with periodic activation functions. In *Proceedings of the 34th International Conference on Neural Information Processing Systems*, NIPS '20, Red Hook, NY, USA, 2020. Curran Associates Inc. ISBN 9781713829546. doi: https://doi.org/10.48550/arXiv.2006.09661.

M. Tancik, P. Srinivasan, B. Mildenhall, S. Fridovich-Keil, N. Raghavan, U. Singhal, R. Ramamoorthi, J. Barron, and R. Ng. Fourier features let networks learn high frequency functions in low dimensional domains. *Advances in neural information processing systems*, 33:7537–7547, 2020. URL https://papers.neurips.cc/paper_files/paper/2020/file/55053683268957697aa39fba6f231c68-Paper.pdf.

Table S3. Hyperparameter configuration for the parameterized PINN solver in Scenario 2 (Autoencoder).

Parameter	Value
**Architecture (PirateNet-inspired)**	
Number of residual blocks (L)	9
Neurons per hidden layer	256
Activation function	Tanh
**Input Embedding**	
Input embedding type	Random Fourier Features
Fourier feature scale	5.0
Random weight factorization	$\mu = 1.0, \sigma = 0.1$
**Optimizer**	
Optimizer type	Adam Kingma and Ba [2017]
Adam β_1	0.9
Adam β_2	0.999
Adam ϵ	10^{-8}
**Learning Rate Schedule**	
Initial learning rate	10^{-3}
Decay schedule	Exponential Decay
Decay rate	0.9
Decay steps	10,000
Warmup steps	5000
**Training**	
Training steps (Stage 1)	2.5×10^4
Training steps (Stage 2)	3×10^5
Batch size	4,096
Collocation points per batch	N_{pde} = 4,096
Boundary points per batch	N_{bc} = 2,048
**Loss Weighting**	
Weighting scheme	Bounded GradNorm
footnote GradNorm bounds [min, max]	[0.02, 50.0]
Initial loss weights (w_{pde}, w_{bc})	(1.0, 1.0)

A. Vaswani, N. Shazeer, N. Parmar, J. Uszkoreit, L. Jones, A. N. Gomez, Ł. Kaiser, and I. Polosukhin. Attention is all you need. *Advances in neural information processing systems*, 30, 2017. URL https://proceedings.neurips.cc/paper_files/paper/2017/file/3f5ee243547dee91fbd053c1c4a845aa-Paper.pdf.

S. Wang, Y. Teng, and P. Perdikaris. Understanding and mitigating gradient flow pathologies in physics-informed neural networks. *SIAM Journal on Scientific Computing*, 43(5):A3055–A3081, Jan. 2021. ISSN 1095-7197. doi: 10.1137/20m1318043. URL http://dx.doi.org/10.1137/20m1318043.

S. Wang, B. Li, Y. Chen, and P. Perdikaris. Piratenets: Physics-informed deep learning with residual adaptive networks. *Journal of Machine Learning Research*, 25(402):1–51, 2024. URL http://jmlr.org/papers/v25/24-0313.html.
